# Functional Comparison of Innate Immune Signaling Pathways in Primates

**DOI:** 10.1371/journal.pgen.1001249

**Published:** 2010-12-16

**Authors:** Luis B. Barreiro, John C. Marioni, Ran Blekhman, Matthew Stephens, Yoav Gilad

**Affiliations:** 1 Department of Human Genetics, University of Chicago, Chicago, Illinois, United States of America; 2 Department of Statistics, University of Chicago, Chicago, Illinois, United States of America; Georgia Institute of Technology, United States of America

## Abstract

Humans respond differently than other primates to a large number of infections. Differences in susceptibility to infectious agents between humans and other primates are probably due to inter-species differences in immune response to infection. Consistent with that notion, genes involved in immunity-related processes are strongly enriched among recent targets of positive selection in primates, suggesting that immune responses evolve rapidly, yet providing only indirect evidence for possible inter-species functional differences. To directly compare immune responses among primates, we stimulated primary monocytes from humans, chimpanzees, and rhesus macaques with lipopolysaccharide (LPS) and studied the ensuing time-course regulatory responses. We find that, while the universal Toll-like receptor response is mostly conserved across primates, the regulatory response associated with viral infections is often lineage-specific, probably reflecting rapid host–virus mutual adaptation cycles. Additionally, human-specific immune responses are enriched for genes involved in apoptosis, as well as for genes associated with cancer and with susceptibility to infectious diseases or immune-related disorders. Finally, we find that chimpanzee-specific immune signaling pathways are enriched for HIV–interacting genes. Put together, our observations lend strong support to the notion that lineage-specific immune responses may help explain known inter-species differences in susceptibility to infectious diseases.

## Author Summary

We know of a large number of diseases or medical conditions that affect humans more severely than non-human primates, such as AIDS, malaria, hepatitis B, and cancer. These differences likely arise from different immune responses to infection among species. However, due to the lack of comparative functional data across species, it remains unclear how the immune system of humans and other primates differ. In this work, we present the first genome-wide characterization of functional differences in innate immune responses between humans and our closest evolutionary relatives. Our results indicate that “core” immune responses, those that are critical to fight any invading pathogen, are the most conserved across primates and that much of the divergence in immune responses is observed in genes that are involved in response to specific microbial and viral agents. In addition, we show that human-specific immune responses are enriched for genes involved in apoptosis and cancer biology, as well as with genes previously associated with susceptibility to infectious diseases or immune-related disorders. Finally, we find that chimpanzee-specific immune signaling pathways are enriched for HIV–interacting genes. Our observations may therefore help explain known inter-species differences in susceptibility to infectious diseases.

## Introduction

Due to our natural focus on humans, we know of a large number of diseases or medical conditions that affect humans more severely than non-human primates. Examples include progression to AIDS following infection with HIV, progression to malaria following infection with *Plasmodium falciparum*, Alzheimer's disease, cancer, and adverse complications following infection with hepatitis B and C (reviewed in [1], [2]). Differences in susceptibility to infectious agents between humans and other primates might be explained, at least in part, by inter-species differences in immune response to infection. Indeed, a large body of work indicates that immune systems are rapidly evolving. In particular, while very little comparative functional data in primates has been collected, recent genomic scans for signatures of natural selection have reported that genes involved in immunity processes are strongly enriched among targets of positive section in human and chimpanzee [3]–[11].

Immune responses are typically classified as either ‘innate’ or ‘adaptive.’ Historically, the focus of most immunological studies has been on the adaptive response and its hallmarks, namely the generation of a large repertoire of antigen-recognizing receptors and immunological memory. Recently, however, more effort has been expended on understanding the innate immune system, as it became clear that innate immunity is an evolutionarily ancient defense mechanism, which governs the initial detection of pathogens and stimulates the first line of host defense [12]–[15]. Moreover, innate immune responses were shown to play a pivotal role in the development of pathogen-specific humoral and cellular adaptive immune responses, which are mediated by B and T cells [16]–[18].

The recognition of pathogens by the innate immune system is primarily mediated by phagocytic cells (e.g., monocytes, macrophages, and dendritic cells) through germline-encoded receptors, known as pattern recognition receptors (PRRs) [17], [19], [20]. The PRRs recognize conserved molecular features characteristic of the microbial world, commonly referred to as pathogen-associated molecular patterns (PAMPs) [17], [19], [20]. Among the different PRRs, the Toll-like receptor (TLR) family, which comprises 10 functional members in humans, has been the most extensively studied. For example, by stimulating primary cell cultures with different TLR agonists *in vitro* (e.g., references [21]–[23]) and by studying mouse models that lack one or several TLRs (e.g., references [24]–[26]), it has been shown that TLRs can be activated in response to virtually any microbe that invades the host.

Once activated, TLRs play a crucial role in orchestrating the response to pathogenic microbial infections through the induction of two major regulatory programs. First, a universal regulatory response, which can be activated by all TLRs and is triggered by infection with a diverse range of microbes or TLR agonists [21], [22], [27]–[29]. This response has been interpreted as a generic ‘alarm signal’ for infection [22], [28], [29]. Second, individual TLRs can activate regulatory programs that are specific to individual microbial agents [19]. Comparative functional studies of TLR-mediated immune response in primates might therefore shed light on inter-species differences in susceptibility to certain infectious agents. However, at present, there is very little functional data with which one can study the evolution of the immune system in primates.

## Results

In order to study functional differences between the innate immune response of humans and two close evolutionary relatives, chimpanzees (*Pan troglodytes*) and rhesus macaques (*Macaca mulatta*), we stimulated primary monocytes from six individuals from each of the three species with LPS for 4, 12, and 24 hours (see Figure S1 for an illustration of the study design). LPS activates the TLR pathway (specifically, TLR4) and mimics an infection with Gram negative bacteria [16]–[18]. We chose this treatment because LPS, *via* TLR4, activates multiple immune signaling pathways, leading to the induction of both inflammatory and ‘viral-like’ responses [19]. Additionally, the stimulation of immune cells with LPS was shown to result in a very similar regulatory response (87% overlap) to the response to infection with a live bacteria such as *E. coli* [22].

To confirm that the LPS treatment activated TLR4-mediated immune responses, we used quantitative PCR to estimate the induction levels of three inflammatory cytokines (*IL6*, *IL1β*, and *TNF*). In all samples (from all individuals at all time points), levels of the three inflammatory cytokines were significantly higher following stimulation with LPS (Figure S2). However, we noticed that the quantitative responses to the treatment in the chimpanzee samples were lower than those of the human and rhesus macaque samples. This observation probably reflects a technical difficulty in culturing chimpanzee primary monocytes without inducing a general stress response, which results in the attenuation of the quantitative response to further stimuli (see Materials and Methods for more details). In what follows, we therefore focus primarily on qualitative rather than quantitative differences between individuals and species in the regulatory response to stimulation with LPS.

### LPS-mediated immune responses in primates

To estimate and compare gene expression levels in samples from multiple species, we used a multispecies microarray, which includes orthologous probes from human, chimpanzee, and rhesus macaque for 18,109 genes [30]. Following processing and normalization of the array data, we used a gene-specific linear mixed-effect model (see Materials and Methods) to identify inter-species differences in the regulatory response to stimulation with LPS (the ‘treatment’). To minimize the number of falsely identified differences across species, we applied two statistical cutoffs for classifying genes as responding to the treatment. Specifically, conditional on observing a treatment effect with high statistical confidence in one species, we assumed that a treatment effect likely occurred in other species as well, and relaxed the statistical cutoff for the classification of such secondary observations (see Materials and Methods for more details and the specific statistical cutoffs used). This procedure minimizes the number of falsely identified inter-species differences that might ultimately arise from incomplete power to identify differences in gene expression levels following the treatment.

**Figure 1:**
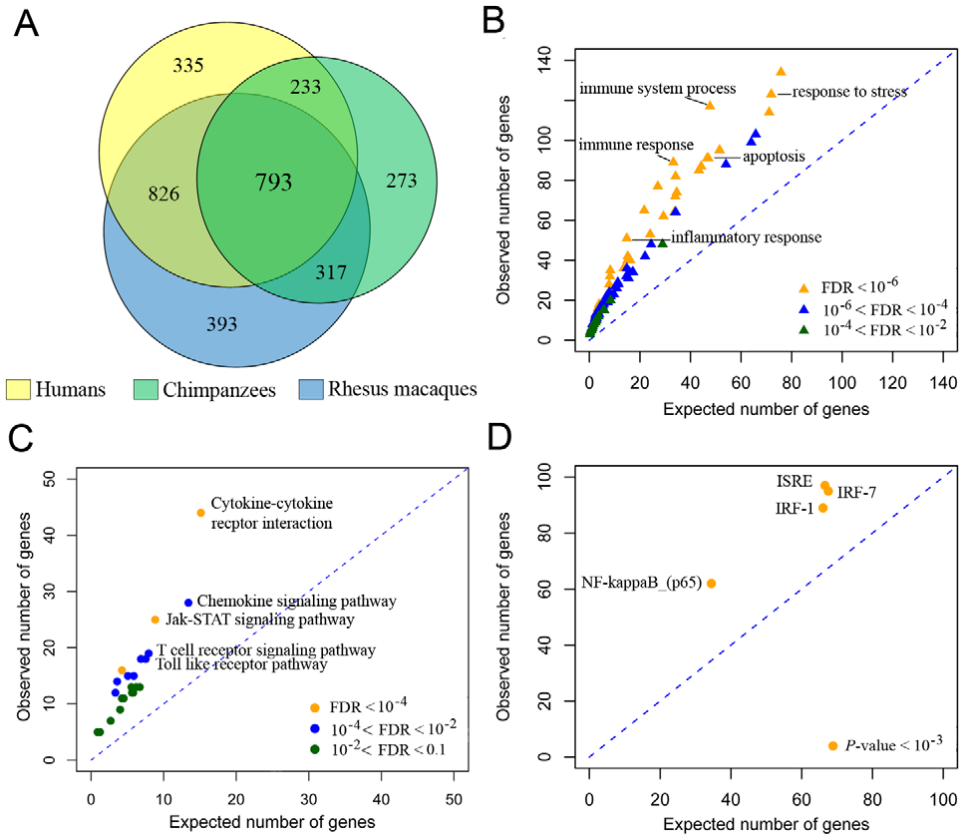
LPS-mediated innate immune response to infection in humans, chimpanzees, and rhesus macaques. (A) Venn-diagram showing the number of genes whose expression levels were altered following stimulation with LPS in humans, chimpanzees and rhesus macaques at any time point (see Figure S4 for data from specific time points). (B) Gene ontology (GO) enrichment analysis using the subset of genes that responded to the treatment in all three species. GO terms related to immunity processes are plotted (see Table S2 for results including all GO terms). (C) KEGG pathway enrichment analysis for genes that responded to the treatment in all three species. (D) Transcription factor binding site enrichment analysis in the promoters of genes that responded to the treatment in all three species (see Table S3 for complete results).

Using this approach, we identified 3,170 genes whose expression levels changed following the treatment in at least one species, at any time point, of which 793 genes responded in all three species (Figure 1A, Table S1, Figure S3). As expected, genes that responded to stimulation with LPS in all three species are enriched with genes involved in immune-related biological processes such as “inflammatory-response” and “cytokine-signaling”, as well as in specific immune-related pathways including the Toll-like receptor pathway, cytokine-cytokine receptor interactions, and the Jak-STAT signaling pathway (FDR for all reported results is <0.01; Figure 1B, 1C, Table S2). Consistent with previous observations in functional studies of the immune system in mice [21], [27], we found that the conserved regulatory response to stimulation with LPS in primates included an enrichment of genes that are likely regulated by the transcription factor NF-kB (*P*<10^−5^) and several interferon regulatory factors (e.g., IRF7 and IRF1; *P*<10^−3^; Figure 1D, Table S3). Put together, these observations clearly demonstrate that the monocytes from all three species responded to the treatment with LPS by engaging TLR4-mediated regulatory pathways [19], leading to the induction of pro-inflammatory and anti-viral immune responses *via* the activation of NF-kB and IRF mediated pathways.

**Figure 2:**
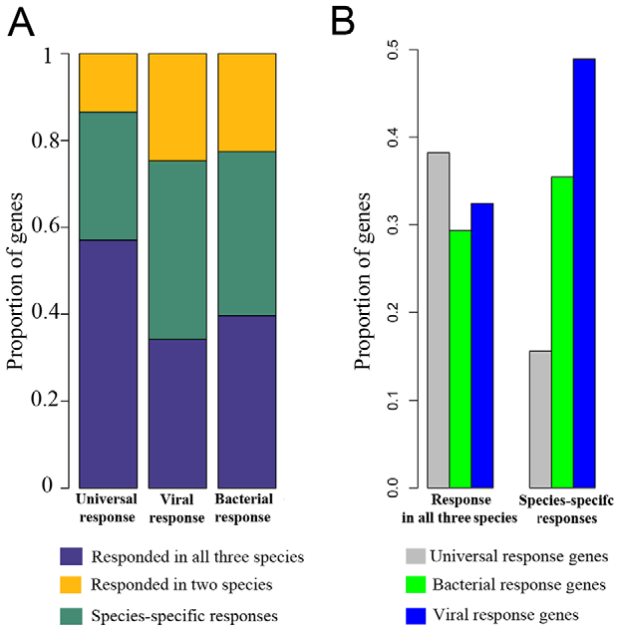
Universal TLR response is more conserved than the immune responses to specific bacterial or viral infections. (A) The proportion of genes that responded to the treatment in all three species, in any two species, or in only one species are plotted for the subsets of genes classified as part of the universal TLR response, the immune responses specific to bacterial infections, or the immune responses specific to viral infections (B) The proportion of genes classified as part of the universal TLR response, the immune responses specific to bacterial infections, or the immune responses specific to viral infections among genes that responded to the treatment in all three species or exclusively in one species. Genes were classified as part of the universal, bacterial, or viral TLR response, based on the findings of Amid and colleagues [21].

To gain further insight into the evolution of LPS-induced immune responses in primates, we classified genes as participating in either the universal regulatory response to infection (which can be triggered by a diverse range of microbes or TLR stimuli [21], [22], [27]), or in the microbial-specific response (which we then further classified as responses to either bacterial or viral infections [21], [22], [27]). Based on these classifications (Table S4), we examined how genes falling into each of the categories responded to infection in humans, chimpanzees, and rhesus macaques. We found that the majority (58%) of genes involved in universal response to infection showed a conserved regulatory response to stimulation with LPS in all three species, compared to only 31% of genes known to respond primarily to either viral or bacterial infection (χ^2^ test, *P*<0.001; Figure 2A). Viewed from a different perspective, we observed that the proportion of genes involved in immune response to viral infections is significantly higher (1.5-fold) among genes that responded to stimulation with LPS in only one of the species, compared with genes that responded to the treatment in all three species (χ^2^ test, *P* = 0.002; Figure 2B). Taken together, our data strongly support the notion that the universal TLR response is mostly conserved across primates and that much of the divergence in immune response is observed in genes that are involved in response to specific microbial and viral agents.

### Characterization of species-specific innate immune responses

**Figure 3:**
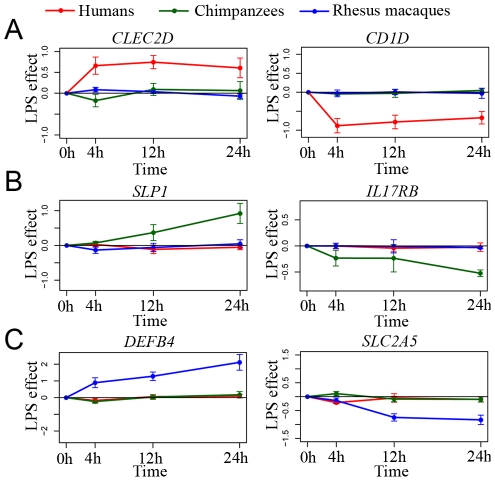
Species-specific responses to infection. Examples of (A) human-specific, (B) chimpanzee-specific, and (C) rhesus-specific immune responses to the treatment. In all panels, the log_2_ fold difference in expression levels (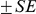) following the treatment (*y*-axis) is plotted for each species at the different time points following infection (*x*-axis).

**Figure 4:**
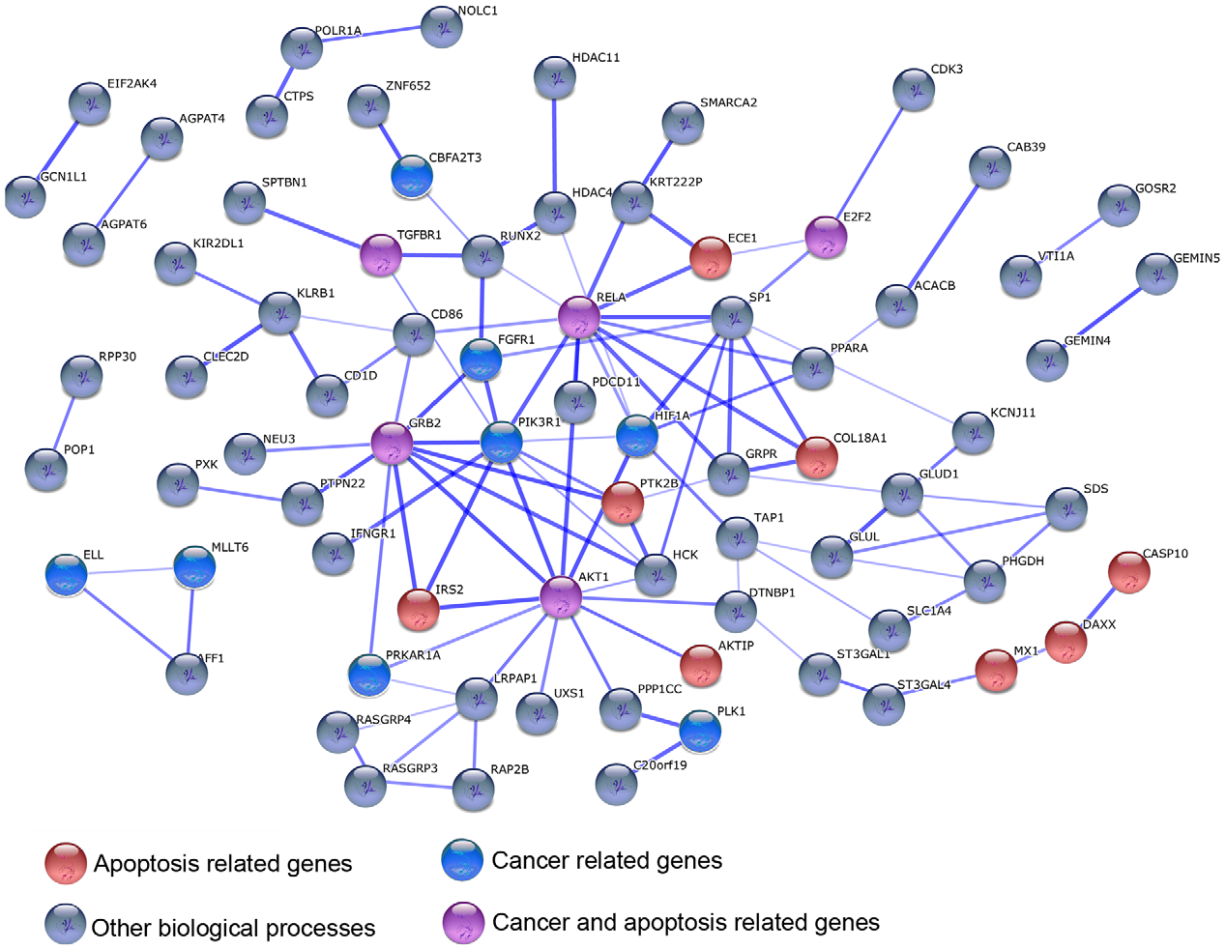
An example of a functional network of genes that responded to stimulation with LPS exclusively in humans. Genes involved in apoptosis and/or cancer pathways are highlighted.

We proceeded by focusing on species-specific immune responses. Using the conservative approach described above, we identified 335, 273, and 393 genes as responding to stimulation with LPS exclusively in the human, chimpanzee, and rhesus macaque monocytes, respectively (see Figure 3A–3C for examples). To characterize these gene sets, we considered functional annotations based on the GO and KEGG databases (Table S5, S6, S7). Somewhat surprisingly, the only significant enrichments (after correction for multiple tests) were observed among the 335 genes that responded to the treatment exclusively in humans. We found that human-specific immune response was enriched for genes in pathways previously associated with cancer (e.g., Chronic myeloid leukemia or prostate cancer; *P*≤3.0×10^−3^, FDR<0.06), the B cell receptor signaling pathway (*P* = 3.2×10^−3^, FDR = 0.06), and pathways related to apoptosis (*P* = 5.0×10^−3^, FDR = 0.07; see Table S5 for a complete list of significant results). Further, by using the STRING database [31] to visualize all known functional interactions between these 335 genes, we found that 151 of the genes in this set (45%) are known to interact with each other – using the default cutoff suggested by STRING to define a functional interaction (Table S8). Applying a more stringent cutoff (a STRING confidence-score higher than 0.7), we identified 78 genes (23%) that interact with each other, in a functional module that is enriched with genes involved in cancer biology and apoptosis pathways (Figure 4, Table S9). In order to obtain further support for interactions across these 78 genes, we used GRAIL, a tool that uses text mining of PubMed abstracts to identify published functional interactions between genes. We found that 43 out of the 78 human-specific immune response genes (55%) had a GRAIL score of *P-*text<0.05, statistically supporting the notion that they have a functional interaction with at least one other gene in the list (only ∼7% of genes are expected to have GRAIL score of *P-*text<0.05 in randomly chosen sets of 78 genes).

**Figure 5:**
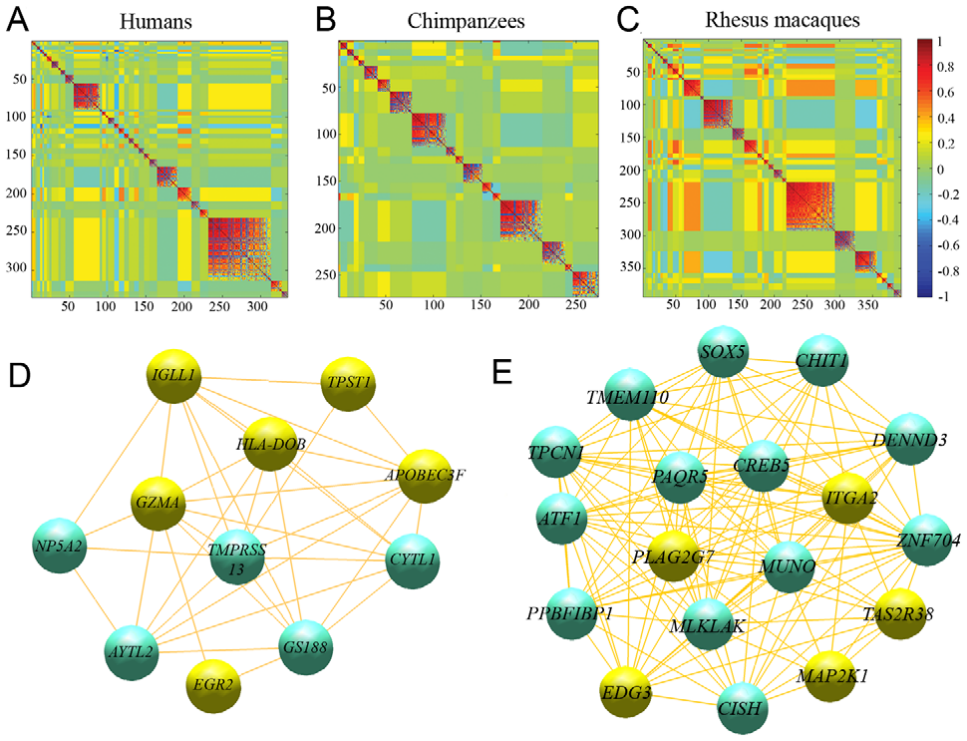
Co-expression regulatory networks. Heatmaps illustrating the correlations of expression profiles for genes responding to stimulation with LPS exclusively in (A) humans, (B) chimpanzees, and (C) rhesus macaques are plotted. Blocks of genes with highly correlated expression profiles correspond to regulatory modules determined by the MMC algorithm. We found 33, 17 and 32 modules in humans, chimpanzees and rhesus macaques, respectively, with an average connectivity (|*r*|) higher than 0.5. In addition to the modules discussed in the text, regulatory modules that merit particular attention include (D) module 13 in chimpanzees, which is significantly enriched for immune response genes (highlighted in yellow), and (E) regulatory module 18 in rhesus macaques, which is significantly enriched for genes involved in immune-related pathways; in particular in MAP kinase signaling pathways (highlighted in yellow), which control a range of cellular activities related to innate immune responses and are particularly important in regulating cytokine gene expression levels and pathways related to programmed cell death [60].

We then considered networks of co-expressed genes (namely genes with coordinated patterns of expression) for each species, to find additional putative modules of interacting genes (see Materials and Methods). We found 33, 17 and 32 regulatory modules in humans, chimpanzees and rhesus macaques, respectively, with an average connectivity (|*r*|) higher than 0.5 (Figure 5, Table S10, S11, S12). Based on 100 random permutations of the gene expression values, we estimated that the number of clusters with |*r*|>0.5 expected by chance alone is 1.28±1.04, 1.16±1.08, or 1.37±1.08, using data from humans, chimpanzees and rhesus macaques, respectively, suggesting that the observed excess of regulatory modules likely describe meaningful biological relationships.

In humans, the largest regulatory module contains 82 genes, which are significantly enriched for several biological processes involved in extracellular matrix remodeling (*P*<0.002; Table S10). The second largest regulatory module is significantly enriched for genes involved in apoptotic pathways (*P*<0.04; Table S10). Interestingly, “apoptosis-related” processes appeared to also be enriched among genes in one of the largest regulatory modules identified in chimpanzees, with 23 genes (Table S11), as well as among three large regulatory modules (>30 genes each) identified in rhesus macaques (Table S12). As the regulatory modules comprise of mutually exclusive sets of genes across the three species (by the nature of the analysis), these observations support the notion that immunological-associated apoptosis mechanisms evolve rapidly in primates.

Finally, we asked whether the observed inter-species differences in immune response might provide insight into the mechanisms underlying differences in susceptibility to infectious diseases between humans and non-human primates. To do so, we considered the subsets of genes that responded to stimulation with LPS exclusively in humans, chimpanzees or rhesus macaques, and examined whether they were enriched for genes previously reported to be associated with immune disorders in humans and/or susceptibility to infectious diseases (see Materials and Methods).

**Figure 6:**
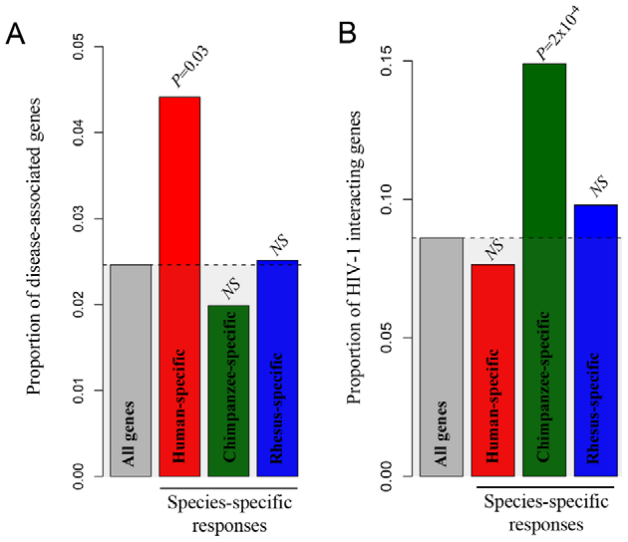
Species-specific immune responses and disease susceptibility. In both panels, the ‘all genes’ category (gray bar) refers to the set of genes that were classified as expressed (based on the array data) in at least one of the conditions (i.e., at any time point in either the treated or untreated samples). (A) The proportions of genes associated with infectious diseases or immune related disorders (*y*-axis) among genes that responded to stimulation with LPS exclusively in humans, chimpanzees and rhesus macaques (x-axis). (B) The proportion of HIV-1 interacting genes (*y*-axis) among the subsets of genes that responded to stimulation with LPS exclusively in each of the three species (x-axis). The observed pattern is robust with respect to the cutoffs used to classify genes as differentially expressed following the treatment (Figure S5).

We found an enrichment of “immune-related-disease-genes” among genes that responded to the treatment with LPS exclusively in humans (χ^2^ test, *P* = 0.03; Figure 6A). Interestingly, we also found that the set of genes that responded to stimulation with LPS exclusively in chimpanzees was enriched with genes that code for host cell proteins known to interact with HIV-1 (Figure 6B; χ^2^ test, *P* = 0.0002). No significant enrichment of HIV-1 interacting genes was observed among genes that responded to stimulation with LPS exclusively in either humans or rhesus macaques. This observation is robust with respect to the specific cutoffs used to classify genes that responded to stimulation in LPS in only one species (Figure S5).

## Discussion

We have performed a genome-wide study of LPS-mediated immune responses in primary monocytes from humans, chimpanzees, and rhesus macaques. Our study design allowed us to characterize conserved innate immune response mechanisms in primates as well as to identify species-specific regulatory responses to stimulation with LPS.

An important difficulty of all studies of gene regulation in primary tissues from primates, apes in particular, is the inability to stage the environment for each of the donor individuals across species. In our study, biological replication within species partially addresses this difficulty, but the possibility that a subset of the observed inter-species differences in gene regulation are due to differences in environments (e.g., diet) across species still exists. An additional difficulty is that most available tools for manipulating cell cultures and performing immune-related assays have not been optimized to work with non-human primate cells. We addressed this issue by performing a large number of quality controls, including the validation of the response to stimulation with LPS in each cell culture by using qPCR. Nevertheless, our observation of systematic differences in the quantitative response to infection across cultures from different species (in particular, from chimpanzees) probably has a technical rather than a biological explanation. For that reason, we chose to draw conclusions primarily based on qualitative differences between species. Thus, the inter-species regulatory differences reported in our study likely provide a lower boundary for the actual number of differences in immune response between humans, chimpanzees and rhesus macaques.

### Conserved innate-immune responses in primates

We identified 793 genes that responded to stimulation with LPS in all three species. As expected, this set of genes was significantly enriched for genes involved in immune responses, and specifically for genes involved in TLR-mediated pathways. Some examples of conserved TLR4-induced immune responses include the strong up-regulation of several pro-inflammatory cytokines, such as *IL-6*, *IL1-β* and tumor necrosis factor (*TNF*), and chemokines, such as *CCL2*, *CCL3* and *CCL4*, whose roles are to recruit other effector cells to the inflammatory site [29], [32]. We also observed a conserved up-regulation of the anti-inflammatory cytokine *IL-10*, probably to control the levels of inflammatory response and avoid tissue damage [29], as well as the up-regulation of several interferon-α inducible genes (e.g., *IFIH1*, *IFIT1*, and *IFIT3*).

Overall, conserved immune responses were enriched for genes whose expression levels are regulated by the transcription factor NF-kB, or by several interferon regulatory factors, which are the master regulators of TLR4-dependent pathways. Interestingly, before infection, the expression levels of many of these master regulators (e.g., *REL*, *NFKB1*, *RELB, IRF2, IRF9*) were different across the three species, while post-infection, their expression converged to practically the same level, regardless of species (Figure S6). This observation suggests that the regulatory response of these key transcription factors likely evolve under strong evolutionary constraints, probably to ensure efficient downstream immune responses.

A known property of the regulatory programs mediated by different TLRs is the activation of both a universal response (shared by all TLRs) as well as a response that is specific to each microbial agent (or TLR ligand) [21], [22], [27]–[29]. We found that the universal TLR response is remarkably more conserved across primates compared to microbial-specific responses. From an evolutionary perspective this observation makes intuitive sense. Indeed, ‘core’ immune responses, which are critical to fight any invading pathogen, are expected to be under stronger evolutionary constraint compared to immune programs that are only important in the presence of specific microbial infections. Consistent with this expectation, our data also support the notion that adaptation of innate immune responses in primates primarily took place at the level of ‘peripheral’ responses, namely, pathogen-specific immune responses.

### Species-specific immune responses

Among genes whose regulation was affected by stimulation with LPS in only one species, we found an enrichment of genes associated with response to viral infections. This observation might reflect the need of the host immune system to frequently devise new defense mechanisms to fight viral infection, as viruses tend to evolve faster than other microbes [33]. We also found that species-specific immune responses are enriched with genes annotated to have a role in apoptotic pathways. Apoptosis is a critical component of successful immune response as infected cells have to be efficiently removed without inciting an inflammatory reaction [34]. Moreover, controlled cell death is used to restore normal cell numbers following clonal expansion of antigen-specific lymphocytes [34]. Consistent with our observation of rapid evolution of the regulation of apoptotic pathways, coding regions of apoptosis-related genes have previously been shown to be rapidly evolving during primate evolution [7], [35]. Put together, these observations suggest that inter-species differences in apoptosis-related immune responses may be adaptive. Although the selective pressures underlying these adaptations are unclear, these observations might help elucidate the basis for important phenotypic differences between humans and non-human primates, such as differences in susceptibility to cancer.

Indeed, cancer incidence in non-human primates is low compared to that observed in humans, even when age is taken into account [1], [2], [36]–[39]. The deregulation of apoptosis has been extensively described as a hallmark of cancers [40]. Thus, while our observation that the human-specific immune response to stimulation with LPS is characterized by a significant enrichment of cancer-related genes is not surprising (because the ‘cancer-related’ and ‘apoptosis-related’ gene sets are not mutually exclusive), it may provide a first step towards understanding the mechanisms underlying the differences in cancer incidence between humans and other primates. For example, we observed that the pro-apoptotic gene *CASP10* was strongly down regulated early after stimulation with LPS, exclusively in humans (Figure S7). Somatic mutations in *CASP10*, as well as reduced expression levels of this gene, were found to be associated with a number of different human cancers [41]–[44]. The observed inter-species differences in the regulation of *CASP10* following infection may therefore be related to differences in the rates of cancer across species. Detailed comparative studies of apoptosis-related regulatory mechanisms in model organisms will be necessary to fully explore the possible connection between the predisposition to cancer and inter-species differences in immune responses.

### Inter-species differences in susceptibility to HIV/AIDS

Genes whose regulation was altered following stimulation with LPS exclusively in humans were enriched with genes known to be associated with susceptibility to infectious diseases or to immune-related diseases. We did not observe such enrichment when we considered the immune responses specific to chimpanzees or rhesus macaques. Our observations make intuitive sense, as we know more about the genes associated with diseases that affect humans than those that affect the two non-human primate species. In other words, it is reasonable to assume that we would have found similar enrichments in chimpanzees and rhesus macaques if we knew more about the genetic basis of infectious and immune-related diseases that primarily affect these two species. Our observations thus underscore the link between species-specific immune responses and susceptibility to infectious disease.

One interesting example is the enrichment of genes known to interact with HIV among genes whose regulation was affected by the stimulation with LPS exclusively in chimpanzee. This observation is intriguing because, unlike humans and rhesus macaques, a large number of studies propose that chimpanzees only rarely develop AIDS following infection with HIV [45]–[47]. This notion has recently been challenged by Keele *et al.* [48], who reported that wild chimpanzees naturally infected with SIVcpz do develop hallmarks of AIDS. The apparently contradictory observations in the literature might be explained by the fact that Keele *et al*. used data collected from an eastern subspecies of chimpanzees (*Pan troglodytes schweinfurthii*), whereas previous observations of increased protection from AIDS, were based on studies with the western chimpanzee subspecies (*Pan troglodytes verus*), the one used in our study. Differences in susceptibility to HIV between sub-species of chimpanzees might be explained by the fact that *Pan troglodytes verus* were physically separated from the two other sub-species prior to systemic infection with the two recombinant monkey viruses of SIVcpz.

Some examples of HIV-interacting gene that responded to stimulation with LPS exclusively in chimpanzees include *ITGB2*(CD18) and *ITGAM*(CD11b), which are the two members of the complement receptor 3 (CR3) that have been shown to play a key role in the infection of dendritic cells by C3-opsonized HIV [49], [50] and the viral transfer to CD4 T cells [50]. Interestingly, these two genes were down-regulated after LPS stimulation, exclusively in chimpanzee monocytes. Another example is the *APOBEC3F* gene, which is one of the most potent inhibitors of HIV replication [51] and was significantly up-regulated in response to stimulation with LPS, only in chimpanzee monocytes. The direction of regulatory change in these cases (namely, the down regulation of a receptor that may be used by the HIV virus, and the up-regulation of a known inhibitor of HIV replication), is consistent with a theoretical mechanism of increased resistance of chimpanzees (or at least *Pan troglodytes verus*) to progression of AIDS. That said, future studies are now required to evaluate if the down-regulation of CR3 and up-regulation of *APOBEC3F* are also observed after infection with HIV (or SIVcpz).

Our observations may reflect an adaptation of the chimpanzee immune system to infection with HIV/SIV or perhaps to other retroviral infection(s). Previous studies of variation at the nucleotide level have reported that genes associated with HIV infection (such as *CD45, APOBEC3G* and *APOBEC3H*) evolved under positive selection in primates [52], particularly after the divergence of humans and chimpanzees [11]. Taken together, our data suggest that regulatory changes occurring specifically in the chimpanzee lineage might explain, at least in part, why chimpanzees tend not to progress to AIDS following infection with HIV/SIV.

More generally, our observations may help to explain other inter-species differences in susceptibility to infectious agents, such as the increased resistance of chimpanzees to certain other viral infections, including hepatitis B and C, and influenza A. Our study, however, is only the first step in characterizing inter-species differences in immune response, in particular because LPS is a general stimulant. We expect future comparative studies in primates to focus on the immune response to different individual infectious agents.

## Materials and Methods

### Ethics statement

This study was conducted according to the principles expressed in the Declaration of Helsinki. An IRB approved consent form was obtained from each human donor. Collection of the non-human primate samples was perform at the Yerkes Primate Center, in a manner that conformed to the animal subject regulatory standards enforced by the Emory University Institutional Animal Care and Use Committee (IACUC approved protocol #028-2009Y).

### Sample collection

We measured gene expression levels in blood monocytes from six humans, six chimpanzees and six rhesus macaques (three males and three females from each species; see Table S13 for details on all samples). Blood samples were collected in BD Vacutainer CPT Cell Preparation Tube (BD, Franklin Lakes, NJ) and peripheral blood mononuclear cells (PBMCs) were purified according to the manufacturer's instructions. Non-human primate blood samples were collected at the Yerkes primate center and human samples were obtained from *Research Blood Components*.

### Monocyte purification and culturing

Blood monocytes from the three species were purified from PBMCs using magnetic cell sorting technology (MACS technology from Miltenyi Biotech). Specifically, monocytes from humans and rhesus macaques were purified by positive selection with magnetic CD14 MicroBeads (Miltenyi Biotech). This method did not work well with the chimpanzee samples (less than 3% of chimpanzee PBMCs were isolated using cell sorting with a CD14 antibody). Instead, monocytes from chimpanzees were purified by depletion of non-monocyte cell types using the “Monocyte Isolation Kit II” (Miltenyi Biotech). Regardless of the method used, the purity of the isolated monocyte population was evaluated by flow cytometry. For the human and rhesus macaque samples we used a fluorochrome-conjugated antibody against monocytes (CD14-FITC; Beckmancoulter). For chimpanzees, we further confirmed the purity of the monocyte population by also using two additional fluorochrome-conjugated antibodies - against B-cells (CD20-PE; BD Bioscience) and T-Cells (CD3-APC BD Bioscience). Regardless of species, only samples with monocyte purity higher than 80% were used in subsequent experiments (Figures S8 and S9). Finally, to further increase the purity of the monocyte-enriched fraction (virtually to 100%), we performed an additional selection step based on the unique capacity of monocytes/macrophages to strongly adhere to the plastic of cell culture dishes. To do so, we cultured the cells overnight (see below for details) and washed the cell culture wells in the morning to retain only adherent cells (i.e., monocytes).

Since we used different methods to purify monocytes in samples from chimpanzees (negative selection) than in samples from humans or rhesus macaques (positive selection), we also performed a control experiment to empirically evaluate to what extent the purification method used affects the ability of the purified cells to respond to LPS stimulation. To do so, we collected whole blood samples from three additional humans, from which we purified monocytes using both a positive and a negative selection method. We then performed the same LPS treatment experiment we applied to the main samples from all three species (described below), and compared differences in response to LPS treatment between monocytes purified by positive selection and monocytes purified by negative selection. As discussed in Text S1 and Figures S15 and S16, we found that the method used to purify the monocytes (negative or positive selection) has only a minimal effect on the measured regulatory response to stimulation with LPS. That said, to be conservative, we excluded from all analyses presented in the manuscript the 192 genes identified as responding to LPS treatment only in monocytes purified by either positive or negative selection.

### Monocyte culture and LPS stimulation

Monocytes were cultured in 24-well cell culture plates (Corning) in serum free media (CTL's test media) at a density of 1 million cells per ml. We used a serum free media to minimize the probability that the monocytes were non-specifically activated as a result of the undefined nature of serum products (e.g., as a result of a mitogenic serum batch). The cells were then stimulated with 1 ug/ml of LPS (Invivogen, Ultrapure LPS, *E. coli* 0111:B4) for 4, 12, and 24 hours. These time points were chosen based on previous observations that the transcription kinetics of immune response to infection can generally be characterized by early, middle, and late phases of response, which can be effectively captured at 4, 12 and 24 hours post infection [22]. All time course experiments were started at the same time of day (∼8am) to prevent the introduction of variation due to differences in circadian rhythm. Untreated cell cultures were kept alongside the stimulated cultures and were harvested at the same time intervals (4, 12, and 24 hours post stimulation).

### RNA extraction and amplification

Total RNA from each cell culture was extracted using RNeasy columns (Qiagen, Valencia, CA). For all samples, RNA quantity was evaluated spectrophotometrically, and the quality was assessed with the Agilent 2100 bioanalyzer (Agilent Technologies Inc, Palo Alto, CA). Only samples with no evidence for RNA degradation (RNA integrity number >8.5) were retained for further experiments. To evaluate the activation of monocytes after stimulation, we used quantitative PCR to test for an over-expression of Tumor-necrosis Factor (*TNF-a*), Interleukine-6 (*IL-6*) and *IL1-β*. inflammatory cytokines that are known to be induced following the activation of TLRs (primer sequences and PCR conditions can be found in Table S14). Only samples for which we observed a significant induction of these cytokines were used in downstream experiments. Once we confirmed that monocytes from all three species responded to the treatment, we performed linear amplifications of the total RNA samples by using in-vitro transcription. Specifically, 400 ng of high-quality total RNA were amplified using the MessageAmp II kit (Ambion). Unlike exponential RNA amplification methods, aRNA amplification has been shown to maintain the relative representation of the starting mRNA population [53], [54] (Figure S10).

### Multi-species microarray

To compare genome-wide gene expression levels between humans, chimpanzees, and rhesus macaques, we hybridized the RNA samples to the multi-species microarray described by Blekhman et al. [30]. This array contains orthologous probes from the three species, thus allowing a comparison of gene expression levels between species without the confounding effects of sequence mismatches on hybridization intensities [30]. The microarray contains probes for 18,109 genes (see Blekhman et al. [30] for a detailed description of the multi-species array). The labeling of the amplified RNA samples and subsequent hybridization to the microarray were performed by Nimblegen. For each individual we hybridized one non-stimulated and one stimulated sample at each of the three time points (4 hours, 12 hours and 24 hours). The total number of arrays analyzed was therefore 108 ( = 3 species ×6 individuals ×6 arrays per individual). Quality control, background correction and normalization of the expression data were performed as previously described [30] (Figure S11 and S12).

### Statistical analysis

All the statistical analyses detailed in this and the following sections were performed using the R statistical environment (http://www.r-project.org).

#### Identifying genes differentially expressed between species

We analyzed data from the 17,231 genes (95% of the genes on the array) that were assayed by at least three orthologous probes across all species. To identify differentially expressed genes between the three species, across time-points and following the treatment, we modeled the expression levels of each gene independently by using a linear mixed-effects model similar to the one described in Blekhman et al. [30]. Specifically, for each of the 17,231 genes, if *y_sroi_* denotes the normalized log_2_ intensity expression value for individual *i* (*i = 1,..6*), from species *s* (*s* = human, chimpanzee or rhesus macaque), measured at probe *r* (*r = 1, …,7*), which is derived from species *o*, we assume that:
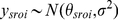
(1)where:
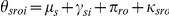



Here, *µ_s_* is a species-specific fixed-effect (representing changes in expression levels across the three species), *π_ro_* is a fixed-effect representing the probe effect for each individual probe within a probe-set and the composition effect of species-specific orthologous probes, and *κ_sro_* is a fixed-effect representing the attenuation of hybridization intensities due to sequence mismatches between species of RNA and a species-specific derived probe, which are different for each individual probe within a probe-set (see reference [30] for more details). The term 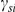 represents a random-effect for individual *i* from species *s*; this effect is assumed to follow a Normal distribution with mean 0 and variance 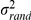. To determine whether a gene was differentially expressed between species, we assessed how well model (1) fitted the data under the following parameterizations of 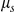:
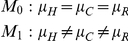



In the null model (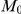) the gene's expression level is assumed to be constant across the three species, while in the alternative model (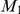) the expression level is allowed to differ between species. We maximized the likelihood under these two parameterizations using an iterated least-squares approach and compared the fit of the models by calculating the likelihood-ratio test statistic. We calculated p-values based on a χ^2^ distribution with two degrees of freedom, and corrected for multiple testing using the FDR approach of Benjamini and Hochberg[55].

#### Identifying genes whose expression level was altered following stimulation with LPS treatment

Due to the paired nature of the study design (untreated and treated cells from the same individual are compared), parameterizations of an extended version of model (1) are difficult to interpret. Instead, we first regressed out probe effects and then used a linear model framework to identify genes whose expression levels have changed following the treatment. To regress out probe effects, we considered data from each time point separately. For each gene, if *y_sroit_* denotes the normalized log_2_ intensity expression value for individual *i* (*i = 1,..6*), from species *s* (*s* = human, chimpanzee or rhesus macaque), from class *t* (*t* = non-stimulated or stimulated) measured at probe *r* (*r = 1,* …,*7*), which is derived from species *o*, we assume that:
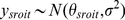
(2)where:
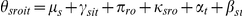



In the above, 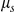, 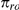, and 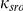 are defined as in the previous section. However, we have added two additional terms to the model: 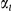 (a treatment effect) and 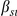 (a treatment-by-species interaction). Further, the parameterization of the random effect has been modified to reflect the incorporation of the treatment effect, such that 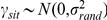. We used a maximum likelihood approach to estimate the parameters in this model, and subsequently calculated a corrected measure of expression, 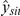 for each individual *i* from each species *s* and for the two classes *t* as: 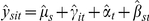 (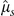 denotes the maximum likelihood estimate of 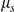 et cetera). We analyzed these corrected measures of expression levels further using gene-wise models. Specifically, for each species separately, if 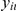 denotes the corrected gene expression level in individual *i* undergoing treatment *t* at the four hour time-point, we fitted the following two models:
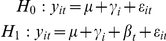



Here, 
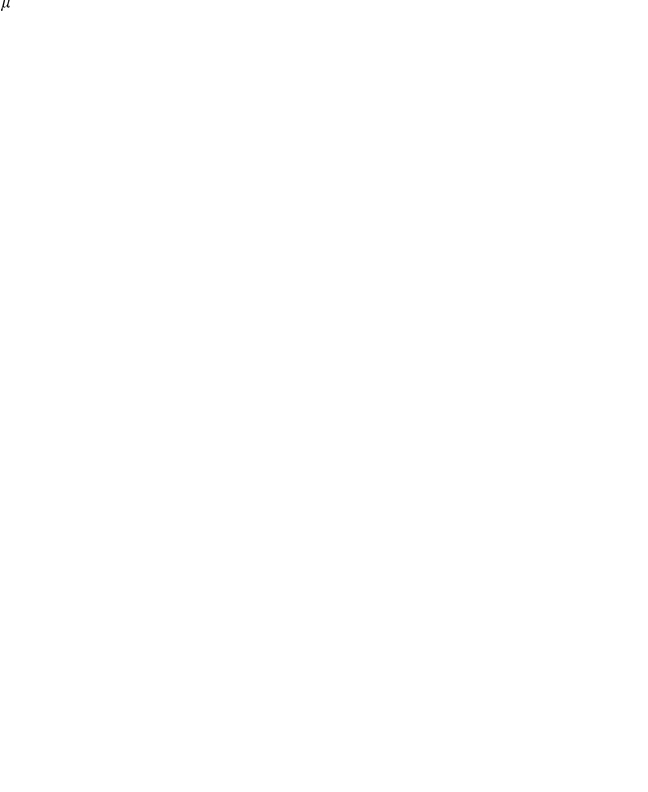
 corresponds to an intercept, 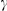 corresponds to an individual effect (shared across treatments), 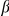 corresponds to a treatment effect and 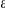 is assumed to follow a normal distribution with mean 0 and variance 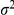. We tested whether there was significant evidence of a difference in expression levels between the treated and un-treated samples by comparing the fit of these models using a likelihood-ratio test statistic. We calculated p-values based on a χ^2^ distribution with one degree of freedom.

Ultimately, our goal was to identify inter-species differences in the regulatory response to stimulation with LPS. To minimize the number of falsely identified differences across species we applied two statistical cutoffs for classifying genes as responding to the treatment. Specifically, as a first step, at each time point, using data from the human and rhesus macaque samples, we initially classified genes as differently expressed following the treatment using an FDR cutoff <0.001, also requiring that the effect size of the treatment was equal or larger than |0.3| (log_2_ scale). For data from the chimpanzee samples, we chose to use different initial cutoffs (an FDR<0.1, and an absolute effect size cutoff of 0.1), as the effect sizes for the regulatory response to the treatment were overall lower (importantly, however, the overlap in lists of responding genes across species is high regardless of the specific cutoff used; Figure S13, Table S15). We then assumed that, conditional on observing a treatment effect with high statistical confidence in one species, a treatment effect likely occurred in other species as well. To classify such secondary observations we used a relaxed cutoff of *P*<0.05. This approach is therefore conservative with respect to identifying differences in immune response across species. While the specific numbers reported in the paper are based on arbitrarily chosen cutoffs, the qualitative conclusions we discuss are robust with respect to the specific choice of cutoffs within a considerable range (Table S16 and Figure S5).

### Gene ontology and pathway enrichment analysis

We used GeneTrail (http://genetrail.bioinf.uni-sb.de) [56] to test for enrichment of functional annotations among different classes of genes (as detailed in the results). In all tests, we used a background set of 13,244 genes, which were classified as expressed (using an absolute log_2_ intensity cutoff of 7.5; Figure S14) in at least one species at one condition; that is, any of the time points for the non-stimulated or stimulated samples. The tests were performed using all GO categories and KEGG pathways. We calculated p-values using a Hyper-geometric distribution, and used the approach of Benjamini and Hochberg [55] to control the false discovery rate.

### Enrichment of transcription factor binding sites

We applied the promoter analysis algorithm PRIMA implemented in the EXPANDER package [57]. Given a target set and a background set of genes, PRIMA identifies transcription factor binding motifs that are significantly more prevalent in the promoter of the target set than in the background set. As background we used all the genes in the array that had at least 3 homologous probes across species and that were expressed in at least one species in one of the six conditions (i.e., any of the three time-point in either treated or non-treated samples).

### Network analysis

We looked for known functional associations between the 335 human-specific LPS response genes using the STRING database (http://string.embl.de/). STRING is a database of both known and predicted protein-protein interactions, which includes direct (physical) and indirect (functional) associations derived from numerous sources, including experimental repositories, computational prediction methods and public text collections [31]. We selected all interactions/associations available for a given node with a combined score greater than 0.7. The scores given in the STRING database define the confidence limit for each described interaction/association. A combined score of 0.7 is recommended as the high stringency criterion by the database authors.

In turn, to identify modules of co-expressed genes, we used the Modulated Modularity Clustering (MMC) algorithm, which seeks community structure in graphical data; that is, a graph of genes connected by edges whose weights reflect the degree to which their transcriptional profiles are correlated (see reference [58] for a detailed description of the method). Co-expression modules were defined using the probe-corrected expression estimates for the set of 335, 273 and 393 genes whose expression levels were altered following the treatment exclusively in humans, chimpanzees and rhesus macaques, respectively.

### Analysis of genes associated with disease

Genes previously reported to be associated with immune disorders and/or susceptibility to any infectious disease were identified using the Genetic Association Database (http://geneticassociationdb.nih.gov/). We downloaded the full GAD dataset on Nov 9, 2009, and parsed the all.xls table, which contains gene-disease associations. We labeled genes as immune-related if they had been associated with diseases for which the ‘disease class’ field in the GAD database was defined as ‘Immune’ or “Infectious diseases”. The list of host genes known to interact with HIV-1 proteins was retrieved from the HIV-1 Human Protein Interaction Database, which catalogues over 1,400 human proteins reported in the scientific literature to participate in HIV-1 to human protein interactions [59].

## Supporting Information

Figure S1 Illustrative representation of the microarray hybridization study design. From each individual we hybridized one non-stimulated (NS) and one LPS stimulated (LPS) sample at each of three time points (4 hours, 12 hours and 24 hours) yielding a total of 6 arrays per individual. The total number of arrays analyzed was therefore 108 ( = 3 species ×6 individuals ×6 arrays per individual).
(0.07 MB DOC)

Figure S2 Representative examples of the induction (y-axis) of TNF-α, IL-6 and IL-1β after stimulation of monocytes with LPS, as measured by real-time PCR in A) humans, B) chimpanzees and C) rhesus macaques, at 4, 12, or 24 hours post treatment (x-axis).
(0.23 MB DOC)

Figure S3 Pairwise comparisons of differences in gene expression levels following the treatment between (a) humans and rhesus macaques, (b) humans and chimpanzees, and (c) rhesus macaques and chimpanzees. Data for genes that were classified as differently expressed following the treatment in both species is plotted in red. As shown, the vast majority of genes responded to the treatment in the same direction, regardless of species.
(0.62 MB DOC)

Figure S4 Venn-diagrams showing the number of genes that responded to the treatments in humans, chimpanzees and rhesus macaques 4- 12- and 24-hours following the treatment.
(0.15 MB DOC)

Figure S5 The proportion of HIV-1 interacting genes (y-axis) among the subsets of genes that responded to stimulation with LPS exclusively in each of the three species. In contrast to the results reported in the main paper, here we used the same cutoffs in all species (i.e., FDR<0.01 and 0.1 absolute fold-change cutoff) to classify genes as differently expressed following the treatment. “all genes” (gray bar) refer to the set of genes that were expressed in at least one of the conditions (i.e., at any time point for either the LPS treated or untreated samples). As can be seen, the pattern we reported in the main paper is robust with respect to the particular choice of the statistical cutoff. We note that the enrichment of HIV-1 interacting genes among chimpanzee-specific responses is also significant when compared to the proportions of HIV-1 interacting genes observed among human- and rhesus macaques-specific immune responses (Chi2 test; P<0.002).
(0.08 MB DOC)

Figure S6 Reduction of inter-species variation in gene expression levels following stimulation with LPS for a number of key transcription factors involved in the regulation of TLR4-dependent pathways. The genes presented in the figure are all differently expressed between species before the treatment (FDR<0.05), yet, following infection (mostly 4 hours after infection) their expression converged to practically the same level, regardless of species (P>0.05). The broken lines are for illustration purposes only.
(0.13 MB DOC)

Figure S7 CASP10 LPS responses in humans, chimpanzees and rhesus-macaques at different time-points post stimulation. The solid lines are for illustration purposes only.
(0.06 MB DOC)

Figure S8 Representative plots of flow-cytometry analyses on total PBMC (left panels) and CD14+ cell fractions (right panels) purified by magnetic cells sorting for A) human and B) rhesus macaque samples.
(0.29 MB DOC)

Figure S9 Plots of flow-cytometry analyses of the different cell fractions in a representative chimpanzee sample, obtained after depletion of non-monocyte populations using MACS technology. Total PMBCs (A), depleted fraction (B) or monocyte enriched fraction (C) were stained with antibodies against CD20 - a marker of B-cells and CD3 - a marker of T-cells (left panels), and CD14 - a marker of monocytes (right panels).
(0.60 MB DOC)

Figure S10 Representative examples of the minimal variance introduced by RNA amplification. The fold induction in expression levels (y-axis) observed 4 hours after stimulation with LPS for three genes (x-axis) is compared between amplified and non-amplified RNA samples.
(0.08 MB DOC)

Figure S11 Boxplots of post normalization gene expression values.
(0.21 MB DOC)

Figure S12 Principal component analysis (PCA) of post normalization array data. As expected based on the known phylogeny of the species, the fist principal component of the data separates humans and chimpanzees from rhesus macaques and the second principal component separates humans from chimpanzees.
(0.09 MB DOC)

Figure S13 Overlap between species (y-axis) in the ranks of genes showing the strongest responses to LPS-stimulation (x-axis).
(0.16 MB DOC)

Figure S14 Cutoff defined to exclude genes that are not expressed. Average intensity (y-axis) is plotted against estimates of the between-individual variance (x-axis). The red line is the cutoff below which genes are likely not to be expressed; hence we excluded these genes from the enrichment analyses.
(0.06 MB DOC)

Figure S15 Impact of the monocytes' purification method on the measured immune responses to LPS stimulation. Correlation between the LPS responses of monocytes purified by positive selection and monocytes purified by negative selection, at (A) 4 hours, (B) 12 hours, and (C) 24 hours after LPS treatment. (D) Venn-diagram showing the number of genes whose expression levels were altered following stimulation with LPS in monocytes purified by positive selection (yellow) and monocytes purified by negative selection (green).
(0.15 MB DOC)

Figure S16 Lack of association between genes that responded to LPS only in monocytes purified by negative section and genes classified as responding to LPS treatment exclusively in chimpanzees. In the y-axis we report the P-value for the enrichment (using a re-sampling procedure) of chimpanzee-specific response genes among genes classified as responding to LPS only in monocytes purified by negative section, using different cutoffs (x-axis). Blue dots refer to a two-cutoff approach as we did in our manuscript. Specifically, condition on observing a gene differently expressed in one of the purification methods at a given cutoff (in the x-axis) we consider that that gene was also differently expressed after LPS treatment in the other purification method at a nominal P-value = 0.05.
(0.07 MB DOC)

Table S1 List of genes analysed in the study.
(10.08 MB XLS)

Table S2 KEGG pathways and Gene Ontology (GO) enrichment analyzes for the set of genes that responded to LPS in all three species. Only the top 100 GO enrichment terms are shown.
(0.16 MB DOC)

Table S3 Results of transcription factor binding sites enrichment analyzes using the promoter sequences of the set of genes that responded to LPS in all three species.
(0.08 MB DOC)

Table S4 List of genes that responded to the LPS treatment in our experiment and that, following the findings from Amit et al., were classified as being part of the universal TLR response, the immune responses specific to bacterial infections, or the immune responses specific to viral infections. As expected, genes classified as part of the universal response are highly enriched for genes annotated as being involved in the toll-like receptor signaling pathway (FDR<10-9; no such enrichment is observed among bacterial- or viral-specific response genes), whereas genes classified as primarily involved in immune responses to viral infections are markedly enriched for genes classified as involved in “response to viruses” (GO term 0009615; FDR = 0.005), and virus-host interaction (GO term 0019048; FDR = 0.04).
(0.29 MB DOC)

Table S5 KEGG pathways enrichment analyzes for the 335 genes that responded to LPS only in humans.
(0.05 MB DOC)

Table S6 KEGG pathways enrichment analyzes for the 273 genes that responded to LPS only in chimpanzees.
(0.03 MB DOC)

Table S7 KEGG pathways enrichment analyzes for the 393 genes that responded to LPS only in rhesus macaques.
(0.03 MB DOC)

Table S8 Functional interaction scores between the set of 335 genes that respond to LPS exclusively in humans. Only interactions with a score higher than 0.4 (the default cutoff in STRING) are shown.
(0.17 MB DOC)

Table S9 Sources of information used to support evidence of a functional interaction between genes that responded to LPS only in humans.
(0.13 MB DOC)

Table S10 Co-expression modules and GO enrichement analyses for Human-specific response genes.
(0.09 MB XLS)

Table S11 Co-expression modules and GO enrichement analyses for Chimpanzee-specific response genes.
(0.08 MB XLS)

Table S12 Co-expression modules and GO enrichement analyses for Rhesus-specific response genes.
(0.10 MB XLS)

Table S13 Details on the samples used in this study
(0.04 MB DOC)

Table S14 Primer sequences and PCR conditions for the genes used to validate the immune response to the treatment.
(0.03 MB DOC)

Table S15 Overlap between the top-ranked genes that responded to the treatment in the different species. We considered the N genes (100, 200, 300, 400, 500, or 1000) showing the largest absolute changes in expression levels following the treatment for each species, and then compared the sets of top ranked genes among species. We report the fold-enrichment for overlap in the paired datasets, relative to that expected by chance (given the number of expressed genes at that time point in each species).
(0.03 MB DOC)

Table S16 Consistent enrichment of apoptosis and cancer related genes among human-specific response genes regardless of the cutoffs (within a considerable range) used to define differently expressed genes.
(0.03 MB DOC)

Text S1 Results from a control experiment testing for possible biases due to the choice of monocyte purification approach.
(0.04 MB DOC)

## References

[1] Varki A (2000). A chimpanzee genome project is a biomedical imperative.. Genome Res.

[2] Varki A, Altheide TK (2005). Comparing the human and chimpanzee genomes: searching for needles in a haystack.. Genome Res.

[3] Chimpanzee Sequencing and Analysis Consortium (2005). Initial sequence of the chimpanzee genome and comparison with the human genome.. Nature.

[4] Bustamante CD, Fledel-Alon A, Williamson S, Nielsen R, Hubisz MT (2005). Natural selection on protein-coding genes in the human genome.. Nature.

[5] Gibbs RA, Rogers J, Katze MG, Bumgarner R, Weinstock GM (2007). Evolutionary and biomedical insights from the rhesus macaque genome.. Science.

[6] Kosiol C, Vinar T, da Fonseca RR, Hubisz MJ, Bustamante CD (2008). Patterns of positive selection in six Mammalian genomes.. PLoS Genet.

[7] Nielsen R, Bustamante C, Clark AG, Glanowski S, Sackton TB (2005). A Scan for Positively Selected Genes in the Genomes of Humans and Chimpanzees.. PLoS Biol.

[8] Arbiza L, Dopazo J, Dopazo H (2006). Positive selection, relaxation, and acceleration in the evolution of the human and chimp genome.. PLoS Comput Biol.

[9] Voight BF, Kudaravalli S, Wen X, Pritchard JK (2006). A map of recent positive selection in the human genome.. PLoS Biol.

[10] Wang ET, Kodama G, Baldi P, Moyzis RK (2006). Global landscape of recent inferred Darwinian selection for Homo sapiens.. Proc Natl Acad Sci U S A.

[11] Barreiro LB, Quintana-Murci L (2010). From evolutionary genetics to human immunology: how selection shapes host defence genes.. Nat Rev Genet.

[12] Hoffmann JA, Kafatos FC, Janeway CA, Ezekowitz RA (1999). Phylogenetic perspectives in innate immunity.. Science.

[13] Janeway CA, Medzhitov R (2002). Innate immune recognition.. Annu Rev Immunol.

[14] Litman GW, Cannon JP, Dishaw LJ (2005). Reconstructing immune phylogeny: new perspectives.. Nat Rev Immunol.

[15] Kimbrell DA, Beutler B (2001). The evolution and genetics of innate immunity.. Nat Rev Genet.

[16] Medzhitov R (2001). Toll-like receptors and innate immunity.. Nat Rev Immunol.

[17] Medzhitov R (2007). Recognition of microorganisms and activation of the immune response.. Nature.

[18] van Duin D, Medzhitov R, Shaw AC (2006). Triggering TLR signaling in vaccination.. Trends Immunol.

[19] Kawai T, Akira S (2010). The role of pattern-recognition receptors in innate immunity: update on Toll-like receptors.. Nat Immunol.

[20] Medzhitov R, Janeway CA (2002). Decoding the patterns of self and nonself by the innate immune system.. Science.

[21] Amit I, Garber M, Chevrier N, Leite AP, Donner Y (2009). Unbiased reconstruction of a mammalian transcriptional network mediating pathogen responses.. Science.

[22] Huang Q, Liu D, Majewski P, Schulte LC, Korn JM (2001). The plasticity of dendritic cell responses to pathogens and their components.. Science.

[23] Nau GJ, Richmond JF, Schlesinger A, Jennings EG, Lander ES (2002). Human macrophage activation programs induced by bacterial pathogens.. Proc Natl Acad Sci U S A.

[24] Poltorak A, He X, Smirnova I, Liu MY, Van Huffel C (1998). Defective LPS signaling in C3H/HeJ and C57BL/10ScCr mice: mutations in Tlr4 gene.. Science.

[25] Qureshi ST, Medzhitov R (2003). Toll-like receptors and their role in experimental models of microbial infection.. Genes Immun.

[26] Wang T, Town T, Alexopoulou L, Anderson JF, Fikrig E (2004). Toll-like receptor 3 mediates West Nile virus entry into the brain causing lethal encephalitis.. Nat Med.

[27] Elkon R, Linhart C, Halperin Y, Shiloh Y, Shamir R (2007). Functional genomic delineation of TLR-induced transcriptional networks.. BMC Genomics.

[28] Jenner RG, Young RA (2005). Insights into host responses against pathogens from transcriptional profiling.. Nat Rev Microbiol.

[29] Ricciardi-Castagnoli P, Granucci F (2002). Opinion: Interpretation of the complexity of innate immune responses by functional genomics.. Nat Rev Immunol.

[30] Blekhman R, Oshlack A, Chabot AE, Smyth GK, Gilad Y (2008). Gene regulation in primates evolves under tissue-specific selection pressures.. PLoS Genet.

[31] Jensen LJ, Kuhn M, Stark M, Chaffron S, Creevey C (2009). STRING 8—a global view on proteins and their functional interactions in 630 organisms.. Nucleic Acids Res.

[32] Wang ZM, Liu C, Dziarski R (2000). Chemokines are the main proinflammatory mediators in human monocytes activated by Staphylococcus aureus, peptidoglycan, and endotoxin.. J Biol Chem.

[33] Pybus OG, Rambaut A (2009). Evolutionary analysis of the dynamics of viral infectious disease.. Nat Rev Genet.

[34] Hildeman D, Jorgensen T, Kappler J, Marrack P (2007). Apoptosis and the homeostatic control of immune responses.. Curr Opin Immunol.

[35] da Fonseca RR, Kosiol C, Vinar T, Siepel A, Nielsen R (2010). Positive selection on apoptosis related genes.. FEBS Lett.

[36] Beniashvili DS (1989). An overview of the world literature on spontaneous tumors in nonhuman primates.. J Med Primatol.

[37] McClure HM (1973). Tumors in nonhuman primates: observations during a six-year period in the Yerkes primate center colony.. Am J Phys Anthropol.

[38] Seibold HR, Wolf RH (1973). Neoplasms and proliferative lesions in 1065 nonhuman primate necropsies.. Lab Anim Sci.

[39] Waters DJ, Sakr WA, Hayden DW, Lang CM, McKinney L (1998). Workgroup 4: spontaneous prostate carcinoma in dogs and nonhuman primates.. Prostate.

[40] Cotter TG (2009). Apoptosis and cancer: the genesis of a research field.. Nat Rev Cancer.

[41] Park WS, Lee JH, Shin MS, Park JY, Kim HS (2002). Inactivating mutations of the caspase-10 gene in gastric cancer.. Oncogene.

[42] Shin MS, Kim HS, Kang CS, Park WS, Kim SY (2002). Inactivating mutations of CASP10 gene in non-Hodgkin lymphomas.. Blood.

[43] Fong PY, Xue WC, Ngan HY, Chiu PM, Chan KY (2006). Caspase activity is downregulated in choriocarcinoma: a cDNA array differential expression study.. J Clin Pathol.

[44] Xu B, Zhou ZG, Li Y, Wang L, Yang L (2008). Clinicopathological significance of caspase-8 and caspase-10 expression in rectal cancer.. Oncology.

[45] Sharp PM, Shaw GM, Hahn BH (2005). Simian immunodeficiency virus infection of chimpanzees.. J Virol.

[46] Novembre FJ, Saucier M, Anderson DC, Klumpp SA, O'Neil SP (1997). Development of AIDS in a chimpanzee infected with human immunodeficiency virus type 1.. J Virol.

[47] Silvestri G (2009). Immunity in natural SIV infections.. J Intern Med.

[48] Keele BF, Jones JH, Terio KA, Estes JD, Rudicell RS (2009). Increased mortality and AIDS-like immunopathology in wild chimpanzees infected with SIVcpz.. Nature.

[49] Bajtay Z, Speth C, Erdei A, Dierich MP (2004). Cutting edge: productive HIV-1 infection of dendritic cells via complement receptor type 3 (CR3, CD11b/CD18).. J Immunol.

[50] Bouhlal H, Chomont N, Requena M, Nasreddine N, Saidi H (2007). Opsonization of HIV with complement enhances infection of dendritic cells and viral transfer to CD4 T cells in a CR3 and DC-SIGN-dependent manner.. J Immunol.

[51] Mbisa JL, Bu W, Pathak VK (2010). APOBEC3F and APOBEC3G inhibit HIV-1 DNA integration by different mechanisms.. J Virol.

[52] Ortiz M, Guex N, Patin E, Martin O, Xenarios I (2009). Evolutionary trajectories of primate genes involved in HIV pathogenesis.. Mol Biol Evol.

[53] Feldman AL, Costouros NG, Wang E, Qian M, Marincola FM (2002). Advantages of mRNA amplification for microarray analysis.. Biotechniques.

[54] Polacek DC, Passerini AG, Shi C, Francesco NM, Manduchi E (2003). Fidelity and enhanced sensitivity of differential transcription profiles following linear amplification of nanogram amounts of endothelial mRNA.. Physiol Genomics.

[55] Benjamini Y HY (1995). Controlling the False Discovery Rate: a Practical and Powerful Approach to Multiple Testing.. Journal of the Royal Statistical Society B.

[56] Backes C, Keller A, Kuentzer J, Kneissl B, Comtesse N (2007). GeneTrail—advanced gene set enrichment analysis.. Nucleic Acids Res.

[57] Ulitsky I, Maron-Katz A, Shavit S, Sagir D, Linhart C (2010). Expander: from expression microarrays to networks and functions.. Nat Protoc.

[58] Stone EA, Ayroles JF (2009). Modulated modularity clustering as an exploratory tool for functional genomic inference.. PLoS Genet.

[59] Fu W, Sanders-Beer BE, Katz KS, Maglott DR, Pruitt KD (2009). Human immunodeficiency virus type 1, human protein interaction database at NCBI.. Nucleic Acids Res.

[60] Dong C, Davis RJ, Flavell RA (2002). MAP kinases in the immune response.. Annu Rev Immunol.

