# Supplementary figures and images for: Functional Comparison of Innate Immune Signaling Pathways in Primates
Source: PLoS Genet. 2010 Dec 16;6(12):e1001249. doi: 10.1371/journal.pgen.1001249 (PMC3002988; doi:10.1371/journal.pgen.1001249)

**
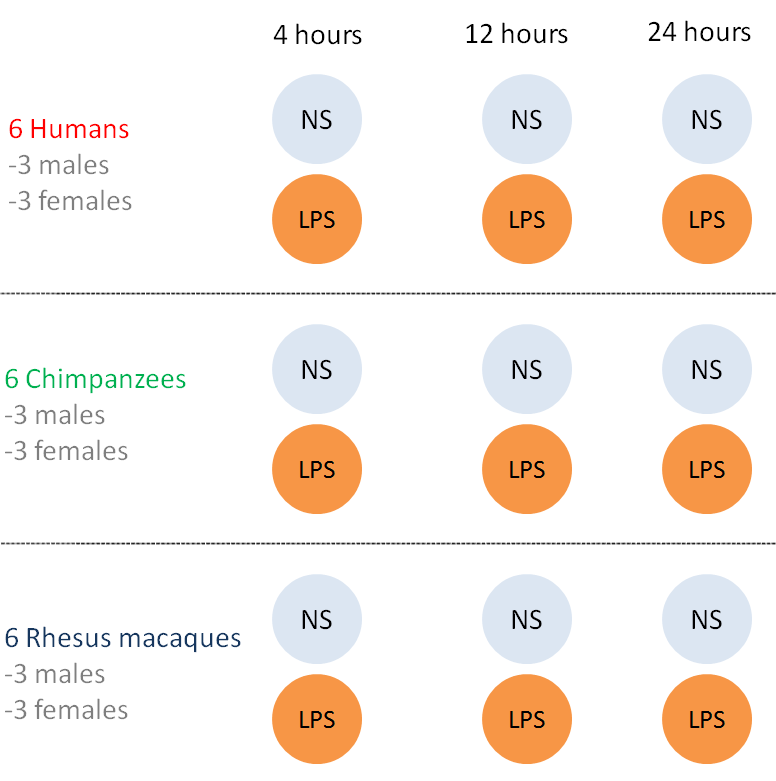
**

Supplement: Figure S1 — Illustrative representation of the microarray hybridization study design. From each individual we hybridized one non-stimulated (NS) and one LPS stimulated (LPS) sample at each of three time points (4 hours, 12 hours and 24 hours) yielding a total of 6 arrays per individual. The total number of arrays analyzed was therefore 108 ( = 3 species ×6 individuals ×6 arrays per individual). (0.07 MB DOC) [file pgen.1001249.s001.doc]

**
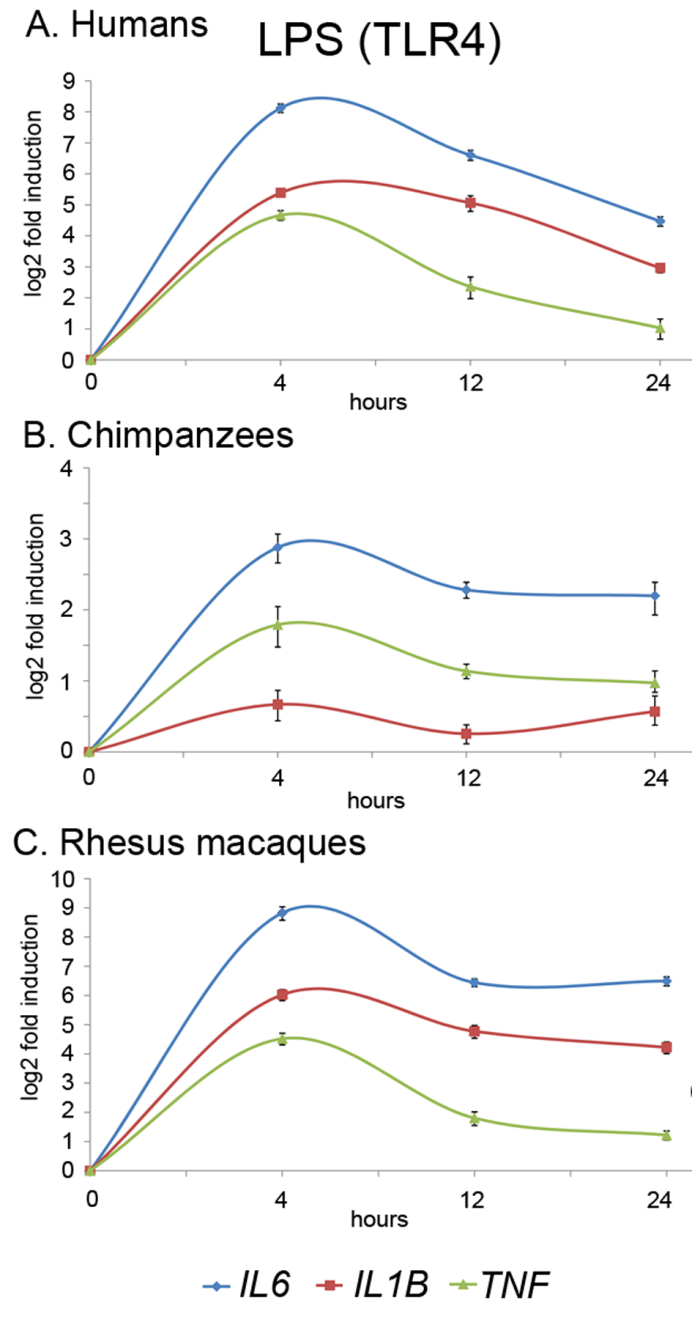
**

Supplement: Figure S2 — Representative examples of the induction (y-axis) of TNF-α, IL-6 and IL-1β after stimulation of monocytes with LPS, as measured by real-time PCR in A) humans, B) chimpanzees and C) rhesus macaques, at 4, 12, or 24 hours post treatment (x-axis). (0.23 MB DOC) [file pgen.1001249.s002.doc]

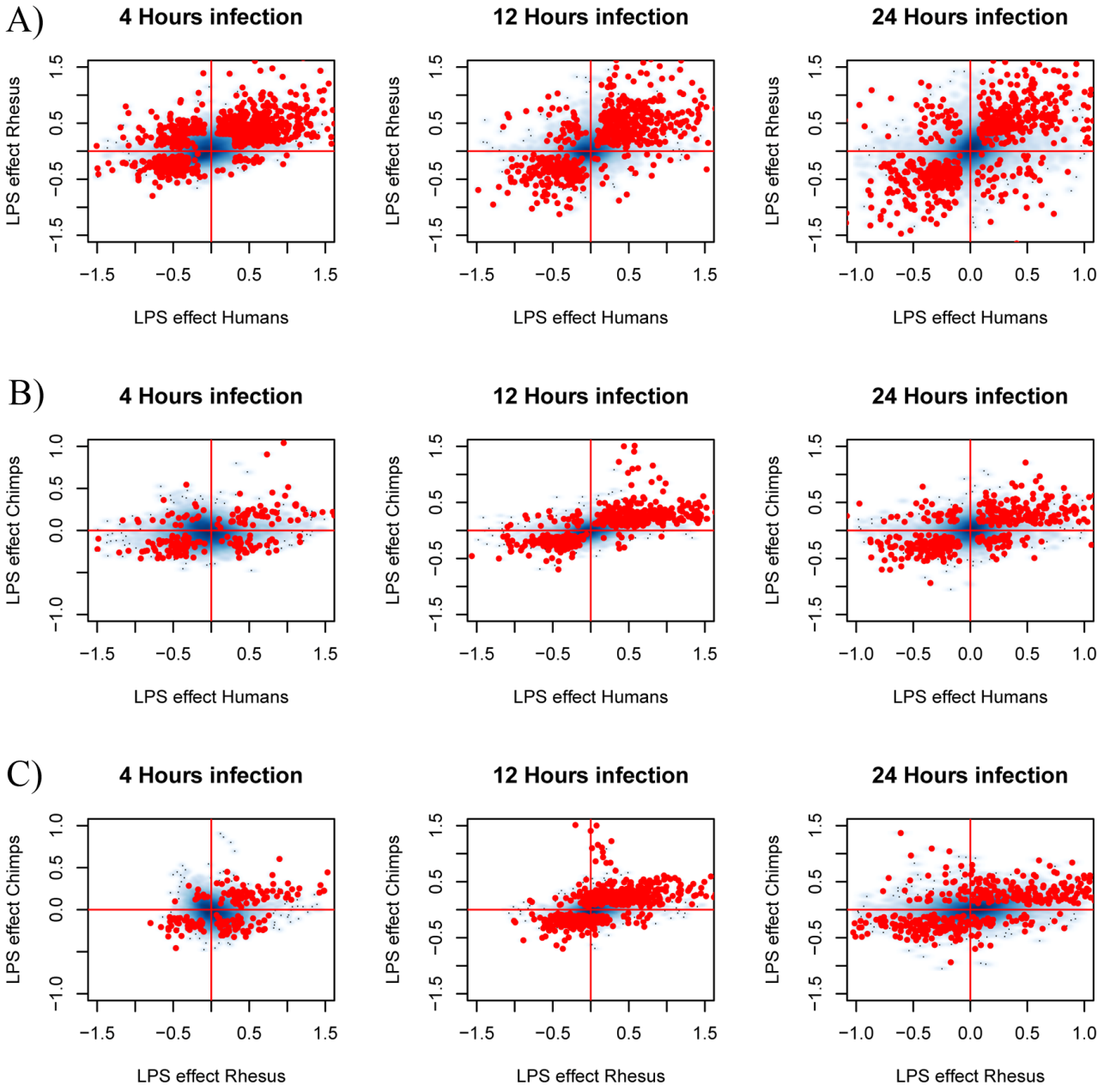

Supplement: Figure S3 — Pairwise comparisons of differences in gene expression levels following the treatment between (a) humans and rhesus macaques, (b) humans and chimpanzees, and (c) rhesus macaques and chimpanzees. Data for genes that were classified as differently expressed following the treatment in both species is plotted in red. As shown, the vast majority of genes responded to the treatment in the same direction, regardless of species. (0.62 MB DOC) [file pgen.1001249.s003.doc]

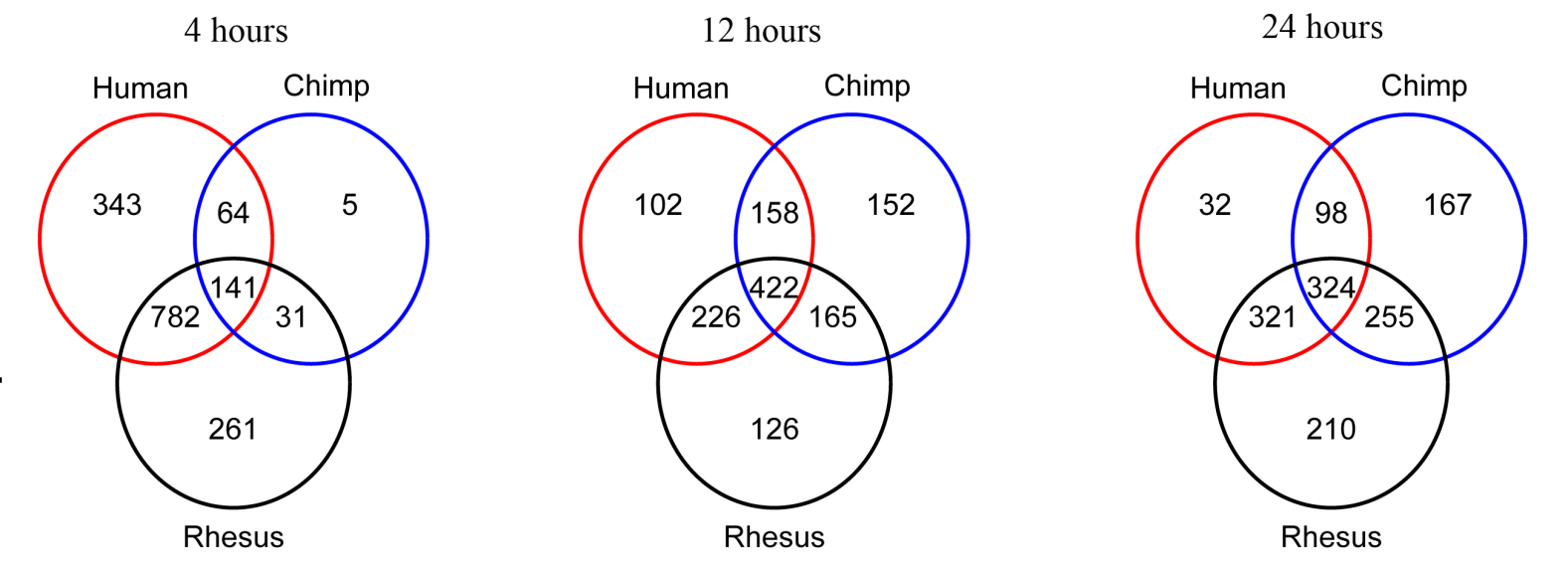

Supplement: Figure S4 — Venn-diagrams showing the number of genes that responded to the treatments in humans, chimpanzees and rhesus macaques 4- 12- and 24-hours following the treatment. (0.15 MB DOC) [file pgen.1001249.s004.doc]

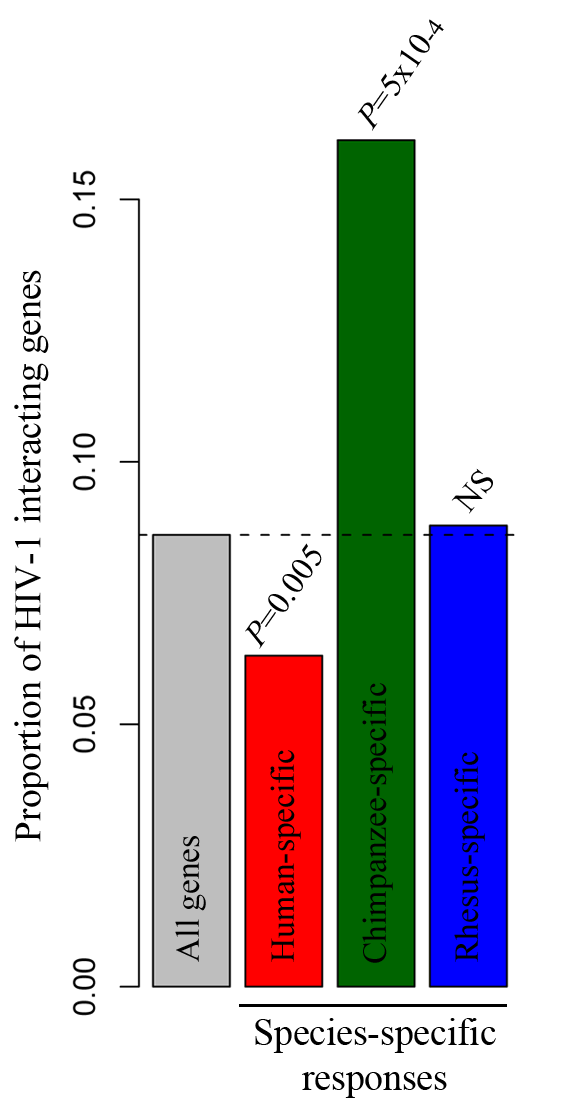

Supplement: Figure S5 — The proportion of HIV-1 interacting genes (y-axis) among the subsets of genes that responded to stimulation with LPS exclusively in each of the three species. In contrast to the results reported in the main paper, here we used the same cutoffs in all species (i.e., FDR<0.01 and 0.1 absolute fold-change cutoff) to classify genes as differently expressed following the treatment. “all genes” (gray bar) refer to the set of genes that were expressed in at least one of the conditions (i.e., at any time point for either the LPS treated or untreated samples). As can be seen, the pattern we reported in the main paper is robust with respect to the particular choice of the statistical cutoff. We note that the enrichment of HIV-1 interacting genes among chimpanzee-specific responses is also significant when compared to the proportions of HIV-1 interacting genes observed among human- and rhesus macaques-specific immune responses (Chi2 test; P<0.002). (0.08 MB DOC) [file pgen.1001249.s005.doc]

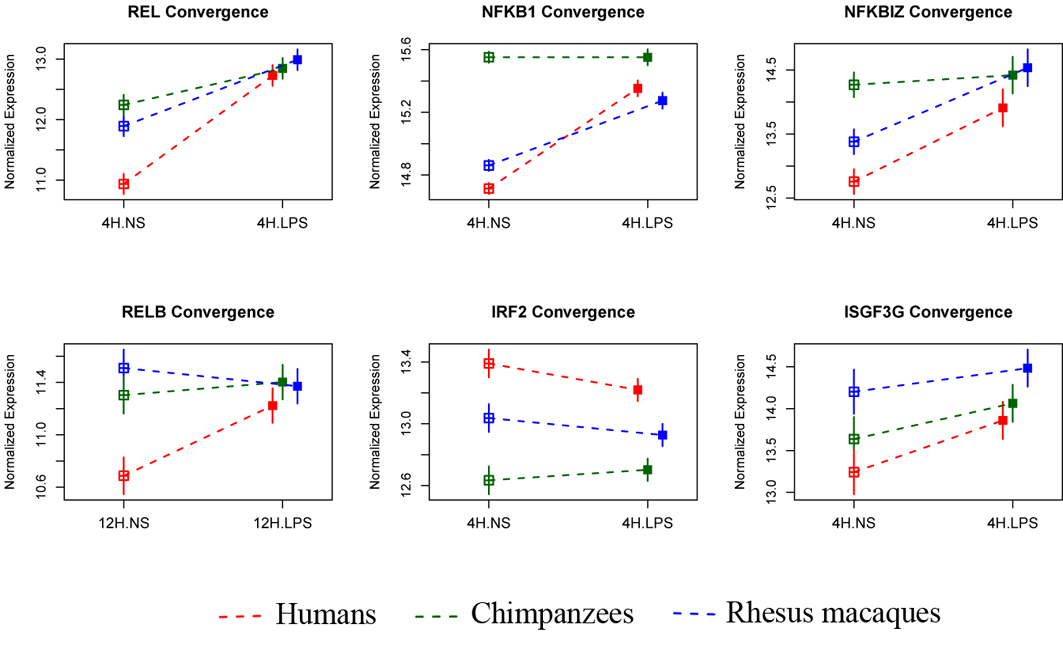

Supplement: Figure S6 — Reduction of inter-species variation in gene expression levels following stimulation with LPS for a number of key transcription factors involved in the regulation of TLR4-dependent pathways. The genes presented in the figure are all differently expressed between species before the treatment (FDR<0.05), yet, following infection (mostly 4 hours after infection) their expression converged to practically the same level, regardless of species (P>0.05). The broken lines are for illustration purposes only. (0.13 MB DOC) [file pgen.1001249.s006.doc]

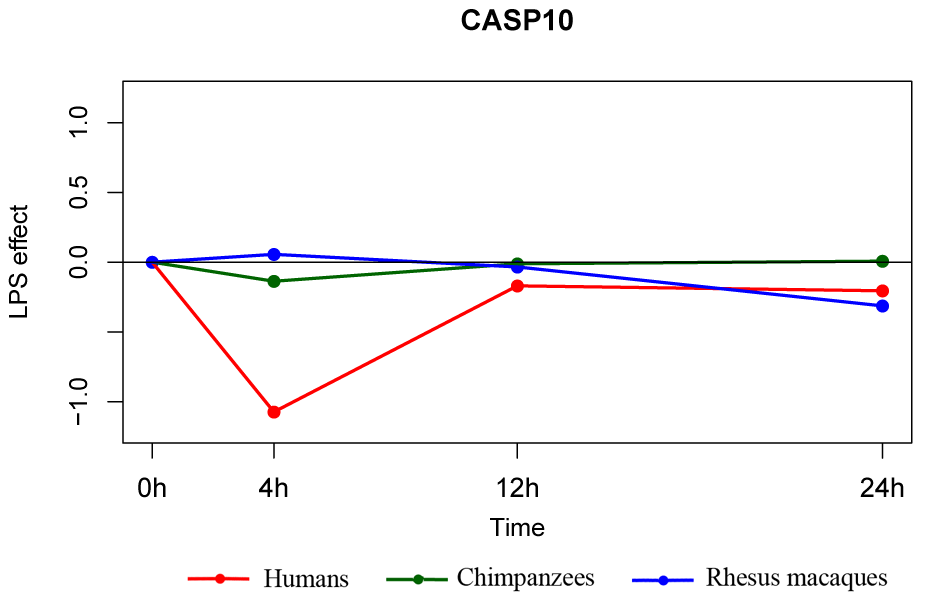

Supplement: Figure S7 — CASP10 LPS responses in humans, chimpanzees and rhesus-macaques at different time-points post stimulation. The solid lines are for illustration purposes only. (0.06 MB DOC) [file pgen.1001249.s007.doc]

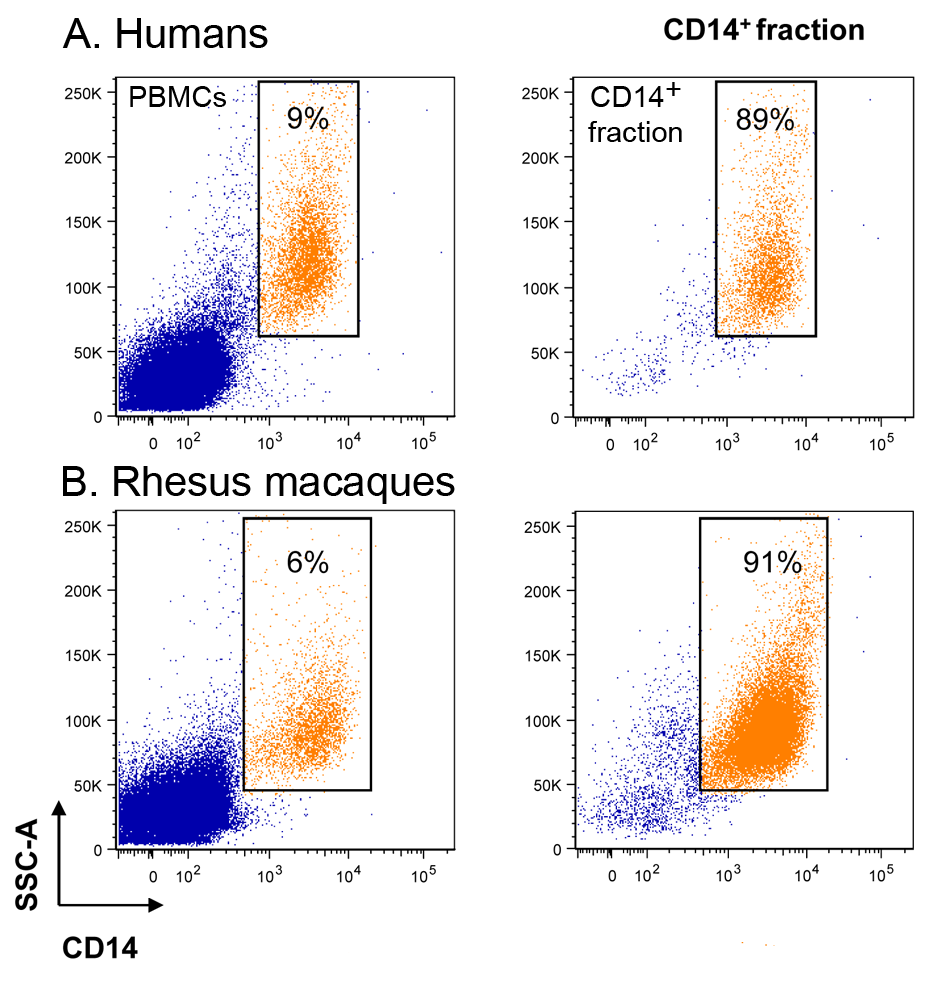

Supplement: Figure S8 — Representative plots of flow-cytometry analyses on total PBMC (left panels) and CD14+ cell fractions (right panels) purified by magnetic cells sorting for A) human and B) rhesus macaque samples. (0.29 MB DOC) [file pgen.1001249.s008.doc]

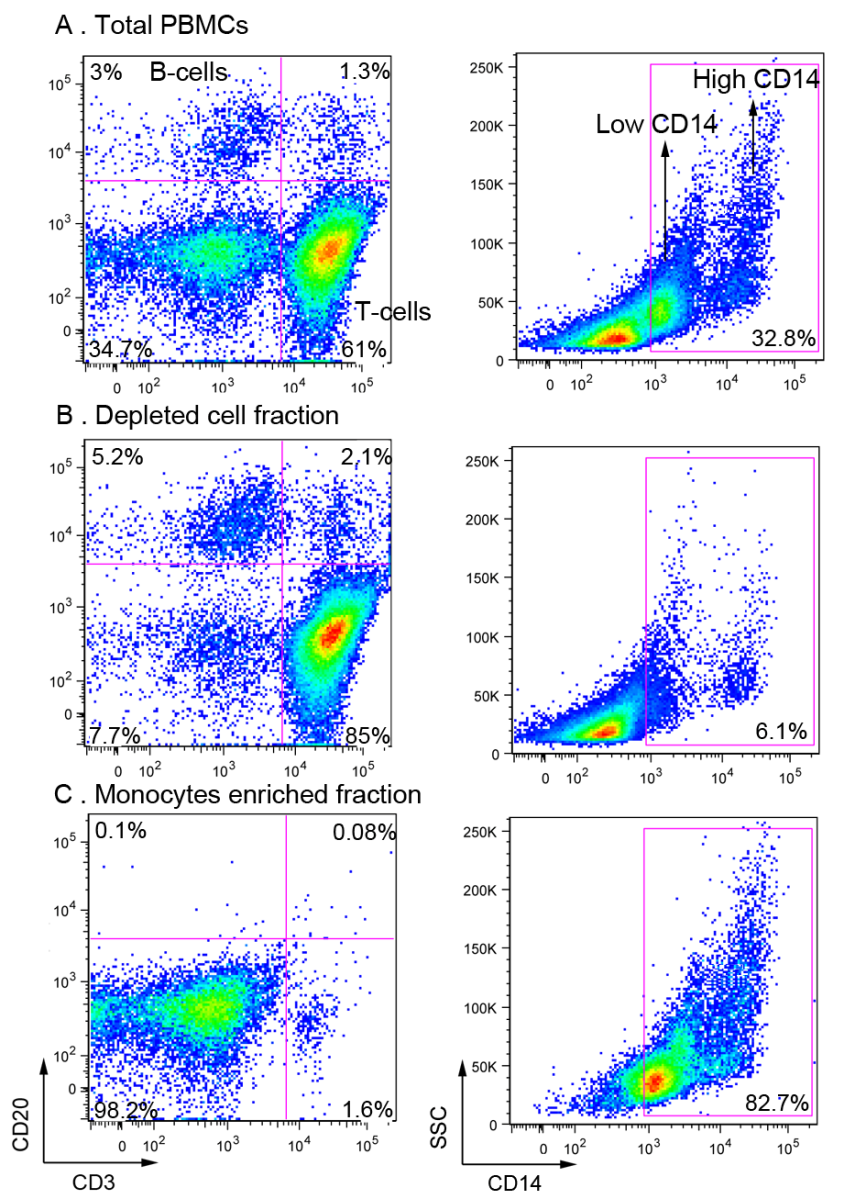

Supplement: Figure S9 — Plots of flow-cytometry analyses of the different cell fractions in a representative chimpanzee sample, obtained after depletion of non-monocyte populations using MACS technology. Total PMBCs (A), depleted fraction (B) or monocyte enriched fraction (C) were stained with antibodies against CD20 - a marker of B-cells and CD3 - a marker of T-cells (left panels), and CD14 - a marker of monocytes (right panels). (0.60 MB DOC) [file pgen.1001249.s009.doc]

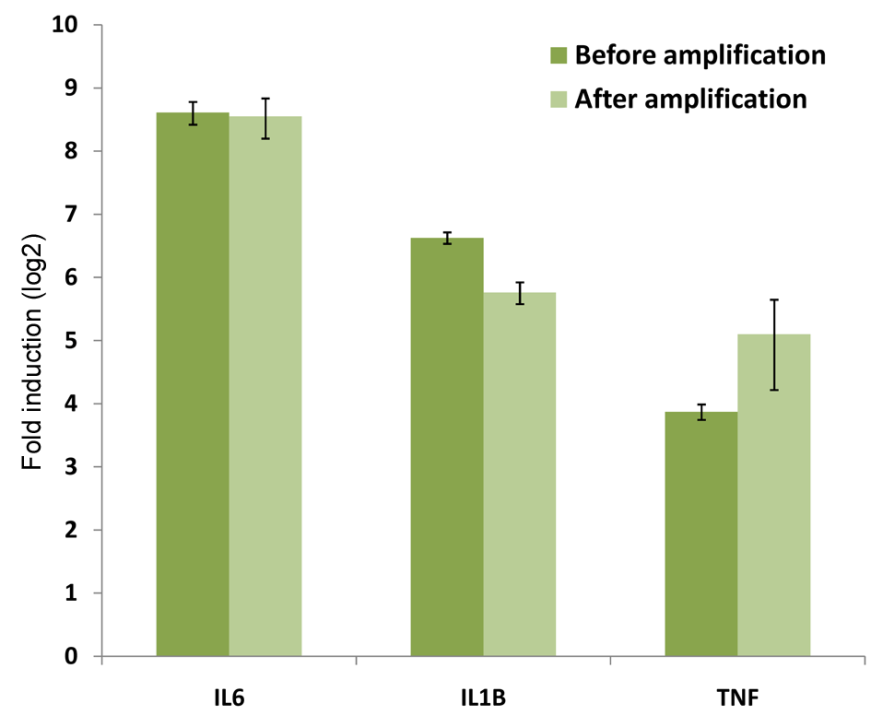

Supplement: Figure S10 — Representative examples of the minimal variance introduced by RNA amplification. The fold induction in expression levels (y-axis) observed 4 hours after stimulation with LPS for three genes (x-axis) is compared between amplified and non-amplified RNA samples. (0.08 MB DOC) [file pgen.1001249.s010.doc]

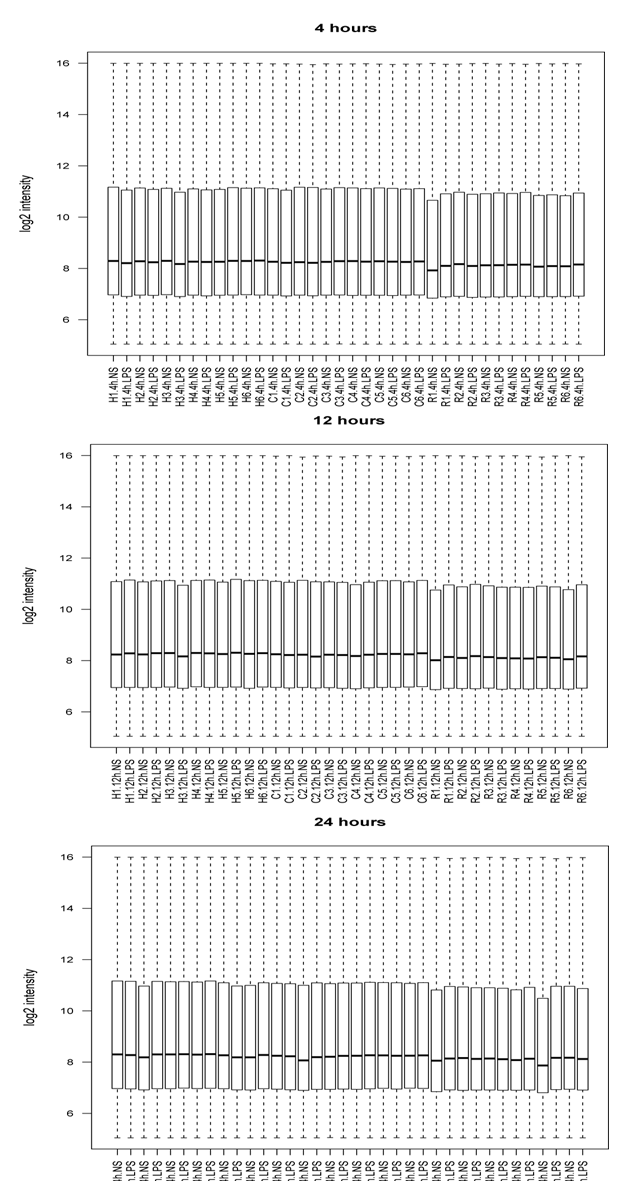

Supplement: Figure S11 — Boxplots of post normalization gene expression values. (0.21 MB DOC) [file pgen.1001249.s011.doc]

**
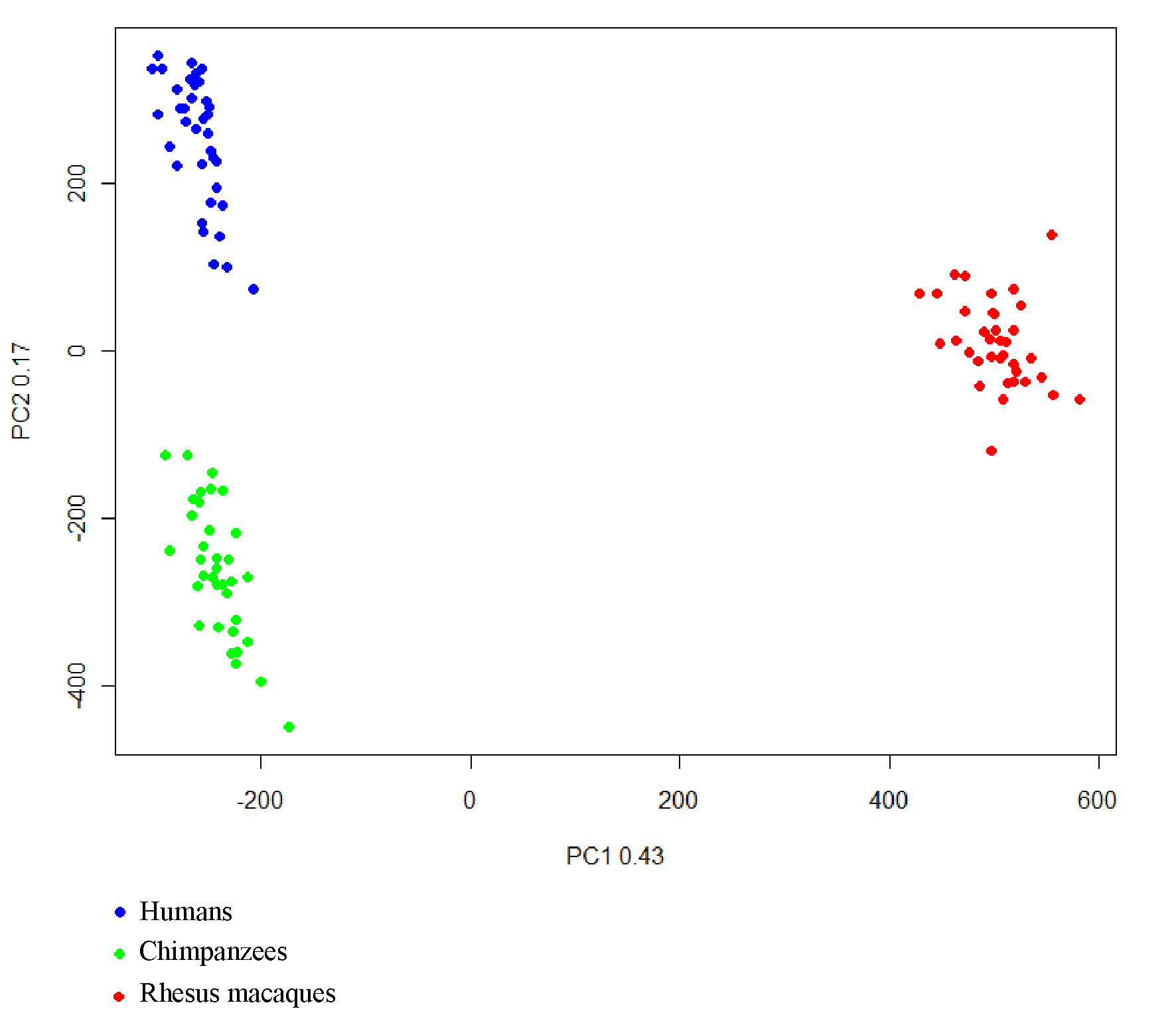
**

Supplement: Figure S12 — Principal component analysis (PCA) of post normalization array data. As expected based on the known phylogeny of the species, the fist principal component of the data separates humans and chimpanzees from rhesus macaques and the second principal component separates humans from chimpanzees. (0.09 MB DOC) [file pgen.1001249.s012.doc]

**
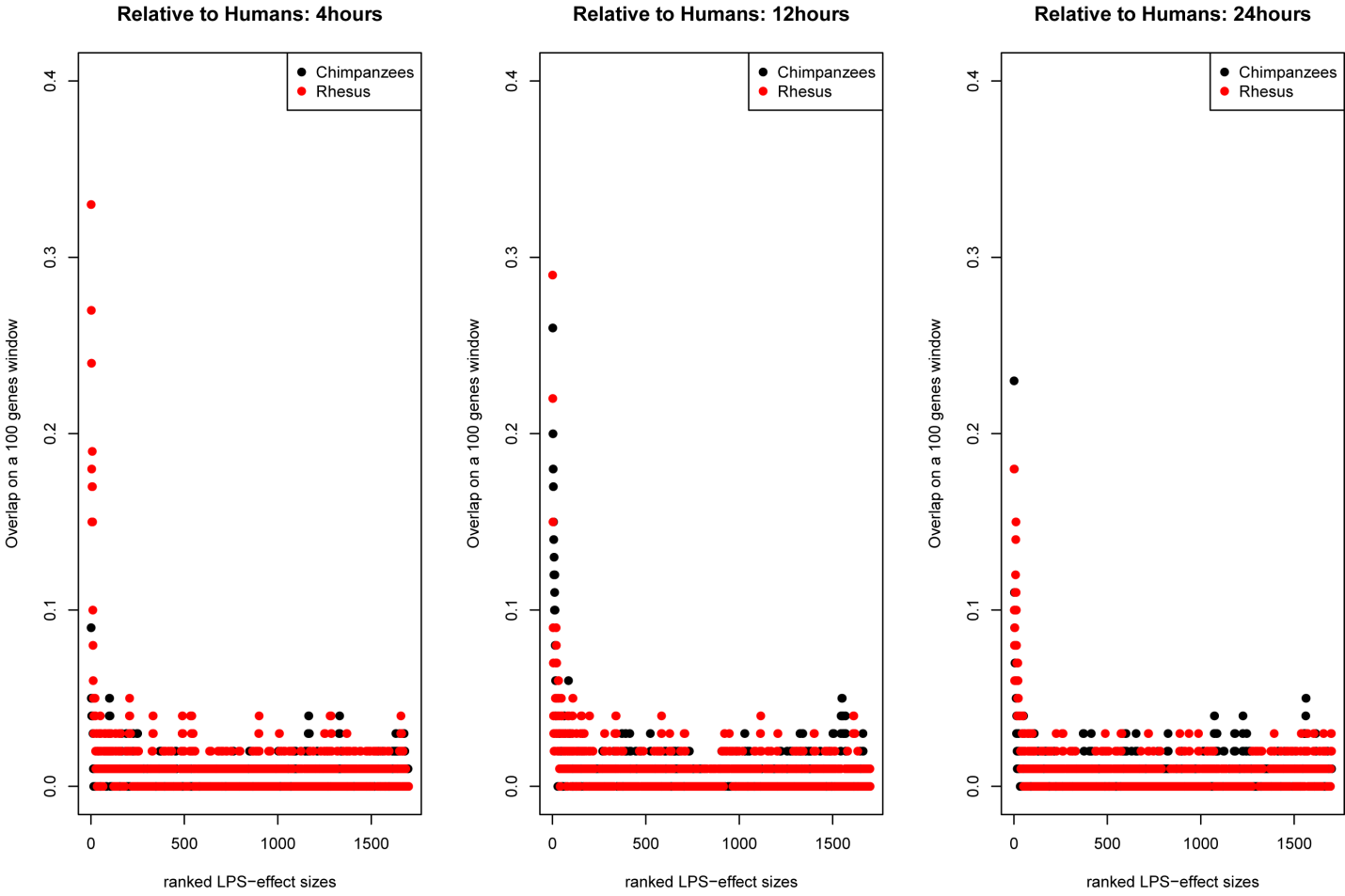
**

Supplement: Figure S13 — Overlap between species (y-axis) in the ranks of genes showing the strongest responses to LPS-stimulation (x-axis). (0.16 MB DOC) [file pgen.1001249.s013.doc]

**
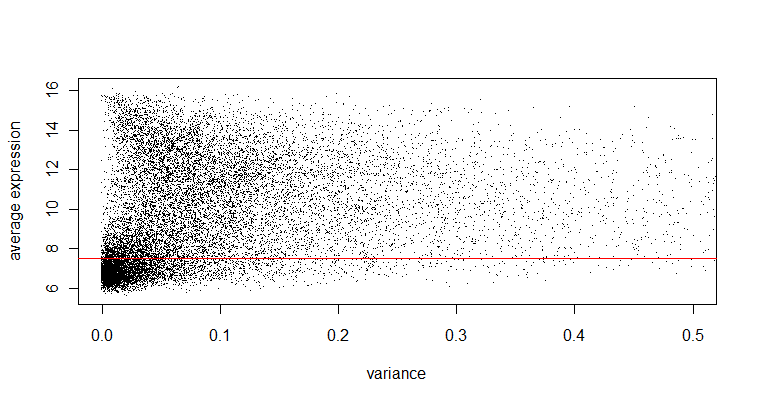
**

Supplement: Figure S14 — Cutoff defined to exclude genes that are not expressed. Average intensity (y-axis) is plotted against estimates of the between-individual variance (x-axis). The red line is the cutoff below which genes are likely not to be expressed; hence we excluded these genes from the enrichment analyses. (0.06 MB DOC) [file pgen.1001249.s014.doc]

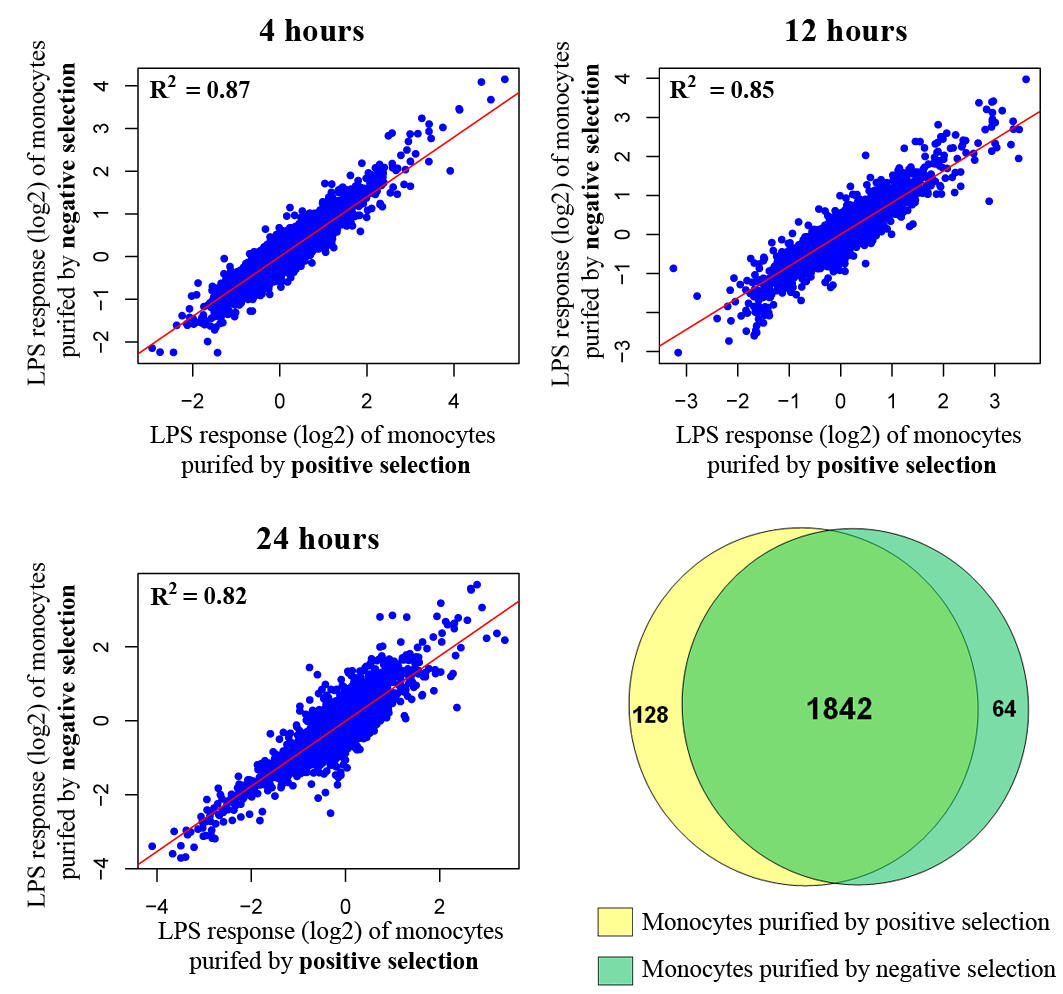

Supplement: Figure S15 — Impact of the monocytes' purification method on the measured immune responses to LPS stimulation. Correlation between the LPS responses of monocytes purified by positive selection and monocytes purified by negative selection, at (A) 4 hours, (B) 12 hours, and (C) 24 hours after LPS treatment. (D) Venn-diagram showing the number of genes whose expression levels were altered following stimulation with LPS in monocytes purified by positive selection (yellow) and monocytes purified by negative selection (green). (0.15 MB DOC) [file pgen.1001249.s015.doc]

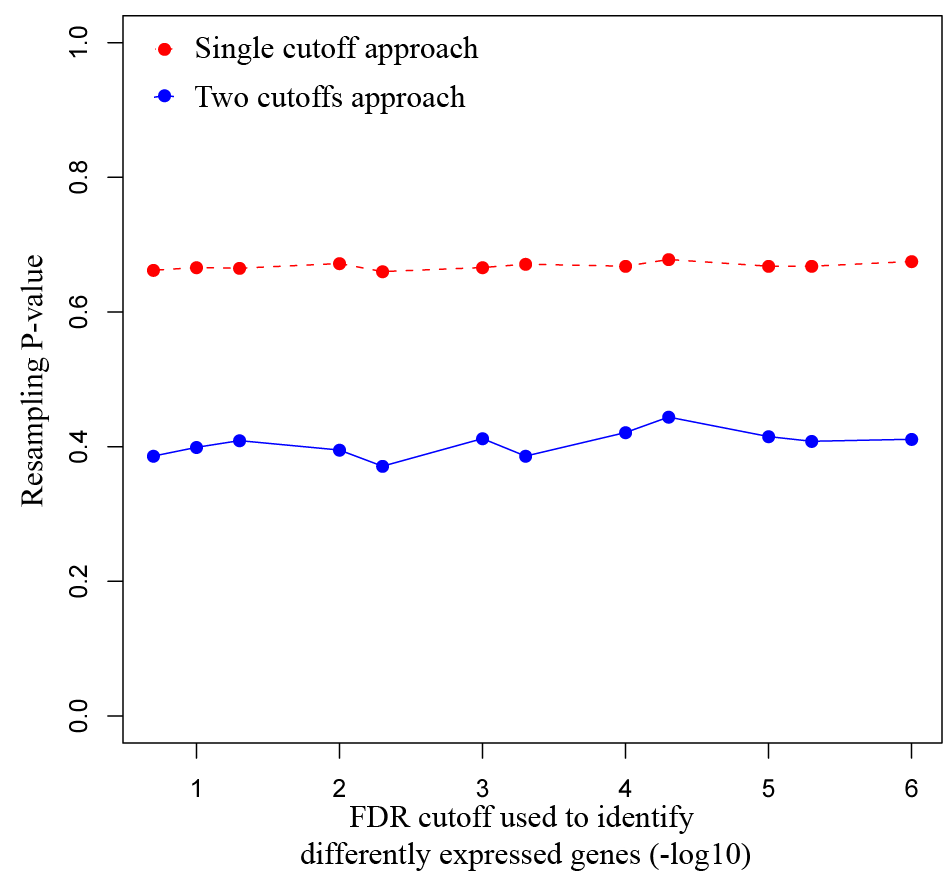

Supplement: Figure S16 — Lack of association between genes that responded to LPS only in monocytes purified by negative section and genes classified as responding to LPS treatment exclusively in chimpanzees. In the y-axis we report the P-value for the enrichment (using a re-sampling procedure) of chimpanzee-specific response genes among genes classified as responding to LPS only in monocytes purified by negative section, using different cutoffs (x-axis). Blue dots refer to a two-cutoff approach as we did in our manuscript. Specifically, condition on observing a gene differently expressed in one of the purification methods at a given cutoff (in the x-axis) we consider that that gene was also differently expressed after LPS treatment in the other purification method at a nominal P-value = 0.05. (0.07 MB DOC) [file pgen.1001249.s016.doc]
