# Supplementary material for: Functional Comparison of Innate Immune Signaling Pathways in Primates
Source: PLoS Genet. 2010 Dec 16;6(12):e1001249. doi: 10.1371/journal.pgen.1001249 (PMC3002988; doi:10.1371/journal.pgen.1001249)
Supplement: Table S2 — KEGG pathways and Gene Ontology (GO) enrichment analyzes for the set of genes that responded to LPS in all three species. Only the top 100 GO enrichment terms are shown. (0.16 MB DOC) [file pgen.1001249.s018.doc]

| **KEGG pathways** | | | | | |
| --- | --- | --- | --- | --- | --- |
| **Subcategory** | **Subcategory alternative name** | **expected** | **observed** | **P-value (raw)er0 P-value (FDR)(** | **P-value (FDR)(** |
| Cytokine-cytokine receptor interaction | 4060 | 15.14 | 44 | 2.15E-11 | 3.01E-09 |
| Jak-STAT signaling pathway | 4630 | 8.84 | 25 | 1.06E-06 | 7.45E-05 |
| NOD-like receptor signaling pathway | 4621 | 4.26 | 16 | 1.90E-06 | 8.86E-05 |
| Malaria | 5144 | 3.60 | 14 | 5.45E-06 | 0.000190899 |
| Cytosolic DNA-sensing pathway | 4623 | 3.36 | 12 | 6.60E-05 | 0.00184876 |
| Leishmaniasis | 5140 | 5.08 | 15 | 9.50E-05 | 0.00197241 |
| Toll-like receptor signaling pathway | 4620 | 6.88 | 18 | 0.000107036 | 0.00197241 |
| Chemokine signaling pathway | 4062 | 13.42 | 28 | 0.000112709 | 0.00197241 |
| T cell receptor signaling pathway | 4660 | 7.94 | 19 | 0.000243623 | 0.00378969 |
| Chagas disease | 5142 | 7.53 | 18 | 0.000359905 | 0.00503867 |
| Amoebiasis | 5146 | 5.89 | 15 | 0.000554395 | 0.00705594 |
| Glycosaminoglycan biosynthesis - keratan sulfate | 533 | 0.90 | 5 | 0.00108819 | 0.0126955 |
| RIG-I-like receptor signaling pathway | 4622 | 4.26 | 11 | 0.00264688 | 0.0264688 |
| Hematopoietic cell lineage | 4640 | 5.57 | 13 | 0.00291633 | 0.0272191 |
| Acute myeloid leukemia | 5221 | 4.50 | 11 | 0.00420097 | 0.0367585 |
| Glycosphingolipid biosynthesis - lacto and neolacto series | 601 | 1.23 | 5 | 0.00537737 | 0.0442842 |
| Apoptosis | 4210 | 6.22 | 13 | 0.00782557 | 0.0608655 |
| B cell receptor signaling pathway | 4662 | 5.65 | 12 | 0.00915159 | 0.0674328 |
| ErbB signaling pathway | 4012 | 5.81 | 12 | 0.0114666 | 0.076444 |
| Fc gamma R-mediated phagocytosis | 4666 | 6.71 | 13 | 0.0146479 | 0.0932139 |
| Type II diabetes mellitus | 4930 | 2.70 | 7 | 0.0153334 | 0.0933337 |
| Adipocytokine signaling pathway | 4920 | 4.01 | 9 | 0.0161798 | 0.0943822 |
| **Gene Ontology** | | | | | |
| **Subcategory** | **Subcategory alternative name** | **expected** | **observed** | **P-value (raw)er0 P-value (FDR)(** | **P-value (FDR)(** |
| defense response | GO:0006952 | 31.89 | 87 | 2.46E-18 | 7.87E-15 |
| inflammatory response | GO:0006954 | 17.43 | 60 | 9.15E-18 | 1.27E-14 |
| immune system process | GO:0002376 | 52.54 | 118 | 1.19E-17 | 1.27E-14 |
| response to wounding | GO:0009611 | 27.72 | 78 | 2.65E-17 | 2.12E-14 |
| immune response | GO:0006955 | 34.29 | 86 | 7.96E-16 | 5.08E-13 |
| signaling | GO:0023052 | 157.10 | 246 | 3.04E-15 | 1.62E-12 |
| cell communication | GO:0007154 | 73.18 | 140 | 8.20E-15 | 3.74E-12 |
| signal transduction | GO:0007165 | 103.18 | 177 | 3.79E-14 | 1.51E-11 |
| cytokine production | GO:0001816 | 11.43 | 42 | 8.29E-14 | 2.94E-11 |
| signal transmission | GO:0023060 | 115.74 | 191 | 1.19E-13 | 3.79E-11 |
| signaling process | GO:0023046 | 116.06 | 191 | 1.55E-13 | 4.50E-11 |
| response to external stimulus | GO:0009605 | 29.49 | 73 | 2.89E-13 | 7.70E-11 |
| apoptosis | GO:0006915 | 55.38 | 110 | 9.48E-13 | 2.29E-10 |
| extracellular space | GO:0005615 | 26.46 | 67 | 1.00E-12 | 2.29E-10 |
| programmed cell death | GO:0012501 | 55.82 | 110 | 1.60E-12 | 3.40E-10 |
| regulation of cytokine production | GO:0001817 | 10.17 | 37 | 3.51E-12 | 7.01E-10 |
| positive regulation of biological process | GO:0048518 | 103.75 | 171 | 4.18E-12 | 7.85E-10 |
| cell death | GO:0008219 | 60.87 | 115 | 7.65E-12 | 1.36E-09 |
| death | GO:0016265 | 60.93 | 115 | 8.19E-12 | 1.38E-09 |
| positive regulation of cellular process | GO:0048522 | 95.35 | 159 | 1.21E-11 | 1.93E-09 |
| cytokine activity | GO:0005125 | 8.21 | 32 | 1.48E-11 | 2.26E-09 |
| response to molecule of bacterial origin | GO:0002237 | 6.50 | 28 | 2.20E-11 | 3.19E-09 |
| cytokine receptor binding | GO:0005126 | 8.84 | 33 | 2.53E-11 | 3.51E-09 |
| cell proliferation | GO:0008283 | 53.42 | 103 | 3.42E-11 | 4.56E-09 |
| response to lipopolysaccharide | GO:0032496 | 5.81 | 26 | 4.42E-11 | 5.64E-09 |
| regulation of cell proliferation | GO:0042127 | 38.83 | 82 | 5.05E-11 | 6.21E-09 |
| response to chemical stimulus | GO:0042221 | 69.65 | 124 | 5.81E-11 | 6.73E-09 |
| regulation of cell activation | GO:0050865 | 9.60 | 34 | 5.90E-11 | 6.73E-09 |
| extracellular region part | GO:0044421 | 34.41 | 75 | 8.17E-11 | 9.00E-09 |
| regulation of apoptosis | GO:0042981 | 44.20 | 89 | 9.18E-11 | 9.78E-09 |
| regulation of cell death | GO:0010941 | 45.02 | 90 | 1.05E-10 | 1.05E-08 |
| signal transducer activity | GO:0004871 | 74.95 | 130 | 1.09E-10 | 1.05E-08 |
| molecular transducer activity | GO:0060089 | 74.95 | 130 | 1.09E-10 | 1.05E-08 |
| regulation of multicellular organismal process | GO:0051239 | 46.66 | 92 | 1.35E-10 | 1.26E-08 |
| regulation of programmed cell death | GO:0043067 | 44.64 | 89 | 1.56E-10 | 1.42E-08 |
| regulation of cell communication | GO:0010646 | 53.04 | 100 | 2.49E-10 | 2.21E-08 |
| positive regulation of lymphocyte proliferation | GO:0050671 | 3.41 | 19 | 2.88E-10 | 2.49E-08 |
| intracellular protein kinase cascade | GO:0007243 | 29.99 | 67 | 3.10E-10 | 2.54E-08 |
| signal transmission via phosphorylation event | GO:0023014 | 29.99 | 67 | 3.10E-10 | 2.54E-08 |
| regulation of response to stimulus | GO:0048583 | 25.45 | 60 | 3.23E-10 | 2.58E-08 |
| signaling pathway | GO:0023033 | 114.61 | 177 | 3.98E-10 | 3.10E-08 |
| positive regulation of mononuclear cell proliferation | GO:0032946 | 3.47 | 19 | 4.15E-10 | 3.16E-08 |
| response to other organism | GO:0051707 | 17.05 | 46 | 5.06E-10 | 3.76E-08 |
| intracellular signal transduction | GO:0035556 | 57.59 | 105 | 5.81E-10 | 4.20E-08 |
| positive regulation of leukocyte proliferation | GO:0070665 | 3.54 | 19 | 5.91E-10 | 4.20E-08 |
| regulation of signal transduction | GO:0009966 | 38.01 | 78 | 6.19E-10 | 4.26E-08 |
| regulation of lymphocyte proliferation | GO:0050670 | 4.74 | 22 | 6.33E-10 | 4.26E-08 |
| response to bacterium | GO:0009617 | 9.91 | 33 | 6.40E-10 | 4.26E-08 |
| regulation of mononuclear cell proliferation | GO:0032944 | 4.80 | 22 | 8.38E-10 | 5.40E-08 |
| regulation of signaling process | GO:0023051 | 38.27 | 78 | 8.46E-10 | 5.40E-08 |
| regulation of defense response | GO:0031347 | 8.02 | 29 | 9.17E-10 | 5.63E-08 |
| regulation of lymphocyte activation | GO:0051249 | 8.02 | 29 | 9.17E-10 | 5.63E-08 |
| regulation of leukocyte activation | GO:0002694 | 9.03 | 31 | 9.53E-10 | 5.74E-08 |
| response to stress | GO:0006950 | 88.65 | 144 | 9.71E-10 | 5.75E-08 |
| regulation of leukocyte proliferation | GO:0070663 | 4.86 | 22 | 1.10E-09 | 6.42E-08 |
| cytokine biosynthetic process | GO:0042089 | 4.48 | 21 | 1.31E-09 | 7.48E-08 |
| cytokine metabolic process | GO:0042107 | 4.55 | 21 | 1.74E-09 | 9.77E-08 |
| positive regulation of cell activation | GO:0050867 | 6.31 | 25 | 1.80E-09 | 9.90E-08 |
| cell activation | GO:0001775 | 18.94 | 48 | 1.87E-09 | 1.01E-07 |
| positive regulation of developmental process | GO:0051094 | 15.98 | 43 | 2.03E-09 | 1.08E-07 |
| lymphocyte proliferation | GO:0046651 | 6.38 | 25 | 2.25E-09 | 1.18E-07 |
| response to biotic stimulus | GO:0009607 | 21.66 | 52 | 2.72E-09 | 1.38E-07 |
| regulation of immune system process | GO:0002682 | 21.03 | 51 | 2.72E-09 | 1.38E-07 |
| mononuclear cell proliferation | GO:0032943 | 6.44 | 25 | 2.80E-09 | 1.40E-07 |
| response to stimulus | GO:0050896 | 152.93 | 218 | 3.00E-09 | 1.47E-07 |
| positive regulation of lymphocyte activation | GO:0051251 | 5.56 | 23 | 3.16E-09 | 1.53E-07 |
| positive regulation of immune system process | GO:0002684 | 13.32 | 38 | 3.43E-09 | 1.61E-07 |
| regulation of signaling pathway | GO:0035466 | 47.42 | 89 | 3.45E-09 | 1.61E-07 |
| leukocyte proliferation | GO:0070661 | 6.50 | 25 | 3.48E-09 | 1.61E-07 |
| regulation of cytokine biosynthetic process | GO:0042035 | 3.98 | 19 | 5.61E-09 | 2.56E-07 |
| regulation of developmental process | GO:0050793 | 35.80 | 72 | 7.13E-09 | 3.21E-07 |
| positive regulation of multicellular organismal process | GO:0051240 | 12.19 | 35 | 1.19E-08 | 5.29E-07 |
| positive regulation of cell proliferation | GO:0008284 | 20.77 | 49 | 1.43E-08 | 6.25E-07 |
| regulation of inflammatory response | GO:0050727 | 3.79 | 18 | 1.55E-08 | 6.62E-07 |
| positive regulation of leukocyte activation | GO:0002696 | 6.00 | 23 | 1.55E-08 | 6.62E-07 |
| leukocyte activation | GO:0045321 | 17.11 | 43 | 1.71E-08 | 7.14E-07 |
| receptor binding | GO:0005102 | 38.71 | 75 | 1.72E-08 | 7.14E-07 |
| biological regulation | GO:0065007 | 314.27 | 387 | 1.79E-08 | 7.32E-07 |
| positive regulation of intracellular protein kinase cascade | GO:0010740 | 11.30 | 33 | 2.06E-08 | 8.21E-07 |
| positive regulation of I-kappaB kinase/NF-kappaB cascade | GO:0043123 | 6.63 | 24 | 2.54E-08 | 1.00E-06 |
| positive regulation of cell death | GO:0010942 | 24.56 | 54 | 3.10E-08 | 1.21E-06 |
| locomotion | GO:0040011 | 27.40 | 58 | 3.74E-08 | 1.40E-06 |
| regulation of cellular process | GO:0050794 | 283.83 | 354 | 3.77E-08 | 1.40E-06 |
| regulation of I-kappaB kinase/NF-kappaB cascade | GO:0043122 | 7.26 | 25 | 3.80E-08 | 1.40E-06 |
| multi-organism process | GO:0051704 | 38.71 | 74 | 3.80E-08 | 1.40E-06 |
| positive regulation of defense response | GO:0031349 | 4.42 | 19 | 3.81E-08 | 1.40E-06 |
| regulation of biological process | GO:0050789 | 297.72 | 368 | 4.33E-08 | 1.57E-06 |
| regulation of response to external stimulus | GO:0032101 | 7.83 | 26 | 4.46E-08 | 1.60E-06 |
| positive regulation of apoptosis | GO:0043065 | 24.18 | 53 | 4.64E-08 | 1.65E-06 |
| regulation of response to stress | GO:0080134 | 15.85 | 40 | 4.81E-08 | 1.69E-06 |
| regulation of intracellular protein kinase cascade | GO:0010627 | 16.48 | 41 | 4.94E-08 | 1.71E-06 |
| positive regulation of programmed cell death | GO:0043068 | 24.25 | 53 | 5.07E-08 | 1.74E-06 |
| I-kappaB kinase/NF-kappaB cascade | GO:0007249 | 10.04 | 30 | 5.27E-08 | 1.79E-06 |
| protein secretion | GO:0009306 | 4.55 | 19 | 6.27E-08 | 2.11E-06 |
| chemotaxis | GO:0006935 | 9.60 | 29 | 6.82E-08 | 2.25E-06 |
| taxis | GO:0042330 | 9.60 | 29 | 6.82E-08 | 2.25E-06 |
| positive regulation of cytokine biosynthetic process | GO:0042108 | 2.90 | 15 | 7.12E-08 | 2.32E-06 |
| extracellular region | GO:0005576 | 66.17 | 109 | 7.73E-08 | 2.49E-06 |
| regulation of localization | GO:0032879 | 32.96 | 65 | 8.53E-08 | 2.72E-06 |
| positive regulation of signal transduction | GO:0009967 | 13.83 | 36 | 1.02E-07 | 3.23E-06 |
