# Supplementary material for: Functional Comparison of Innate Immune Signaling Pathways in Primates
Source: PLoS Genet. 2010 Dec 16;6(12):e1001249. doi: 10.1371/journal.pgen.1001249 (PMC3002988; doi:10.1371/journal.pgen.1001249)
Supplement: Table S3 — Results of transcription factor binding sites enrichment analyzes using the promoter sequences of the set of genes that responded to LPS in all three species. (0.08 MB DOC) [file pgen.1001249.s019.doc]

| **Transcription Factor** | **TRANSFAC matrix** | **expected** | **observed** | ***P-value* (raw)** | **Enrichment factor** |
| --- | --- | --- | --- | --- | --- |
| NF-kappaB_(p65) | M00052 | 34.46 | 62 | 2.09E-06 | 1.799 |
| ISRE | M00258 | 66.58 | 97 | 2.09E-05 | 1.457 |
| IRF-7 | M00453 | 67.52 | 95 | 3.47E-05 | 1.407 |
| HIC1 | M01072 | 219.39 | 267 | 2.63E-04 | 1.217 |
| IRF-1 | M00062 | 66.02 | 89 | 7.43E-04 | 1.348 |
| c-Ets-1 | M00339 | 100.47 | 129 | 0.001 | 1.284 |
| BLIMP1 | M01066 | 92.02 | 121 | 0.001 | 1.315 |
| PEA3 | M00655 | 203.49 | 233 | 0.001 | 1.145 |
| ETF | M00695 | 269.54 | 300 | 0.002 | 1.113 |
| TATA | M00216 | 51.12 | 73 | 0.002 | 1.428 |
| PU.1 | M00658 | 89.19 | 113 | 0.004 | 1.267 |
| ZF5 | M00716 | 214.35 | 254 | 0.004 | 1.185 |
| AP-2 | M00189 | 200.87 | 231 | 0.007 | 1.15 |
| Ik-1 | M00086 | 44.31 | 58 | 0.008 | 1.309 |
| AP-2 | M00915 | 179.10 | 204 | 0.009 | 1.139 |
| Pax-5 | M00143 | 121.89 | 142 | 0.01 | 1.165 |
| TEF-1 | M00704 | 282.32 | 321 | 0.012 | 1.137 |
| Pax-5 | M00144 | 63.81 | 79 | 0.012 | 1.238 |
| AP-4 | M00005 | 79.65 | 101 | 0.014 | 1.268 |
| AP-2 | M00800 | 182.48 | 202 | 0.014 | 1.107 |
| E2F | M00803 | 368.09 | 406 | 0.014 | 1.103 |
| ELF-1 | M00746 | 71.98 | 93 | 0.015 | 1.292 |
| MTF-1 | M00650 | 106.35 | 129 | 0.015 | 1.213 |
| AP-3 | M00690 | 19.22 | 30 | 0.017 | 1.561 |
| Brn-2 | M00145 | 56.65 | 72 | 0.018 | 1.271 |
| MOVO-B | M01104 | 229.80 | 256 | 0.02 | 1.114 |
| Pax-2 | M00486 | 24.19 | 35 | 0.021 | 1.447 |
| IRF1 | M00747 | 102.94 | 119 | 0.025 | 1.156 |
| SRF | M00186 | 27.36 | 38 | 0.025 | 1.389 |
| MAF | M00648 | 75.44 | 90 | 0.026 | 1.193 |
| PPARG | M00515 | 33.72 | 46 | 0.028 | 1.364 |
| NF-kappaB_(p50) | M00051 | 48.00 | 60 | 0.029 | 1.25 |
| p300 | M00033 | 74.31 | 92 | 0.029 | 1.238 |
| AP-2alpha | M00469 | 120.11 | 135 | 0.03 | 1.124 |
| SRF | M00152 | 32.28 | 42 | 0.03 | 1.301 |
| UF1H3BETA | M01068 | 280.47 | 313 | 0.033 | 1.116 |
| Lyf-1 | M00141 | 39.52 | 49 | 0.037 | 1.24 |
| FOXO4 | M00472 | 73.83 | 90 | 0.037 | 1.219 |
| TCF11:MafG | M00284 | 61.63 | 71 | 0.042 | 1.152 |
| GCM | M00634 | 43.24 | 55 | 0.044 | 1.272 |
| SZF1-1 | M01109 | 64.49 | 75 | 0.046 | 1.163 |
| Sp1 | M00196 | 351.38 | 370 | 0.047 | 1.053 |
