# Supplementary material for: Functional Comparison of Innate Immune Signaling Pathways in Primates
Source: PLoS Genet. 2010 Dec 16;6(12):e1001249. doi: 10.1371/journal.pgen.1001249 (PMC3002988; doi:10.1371/journal.pgen.1001249)
Supplement: Table S4 — List of genes that responded to the LPS treatment in our experiment and that, following the findings from Amit et al., were classified as being part of the universal TLR response, the immune responses specific to bacterial infections, or the immune responses specific to viral infections. As expected, genes classified as part of the universal response are highly enriched for genes annotated as being involved in the toll-like receptor signaling pathway (FDR<10-9; no such enrichment is observed among bacterial- or viral-specific response genes), whereas genes classified as primarily involved in immune responses to viral infections are markedly enriched for genes classified as involved in “response to viruses” (GO term 0009615; FDR = 0.005), and virus-host interaction (GO term 0019048; FDR = 0.04). (0.29 MB DOC) [file pgen.1001249.s020.doc]

| **TLR Universal response** | | **Bacterial response genes** | | **Viral response genes** | |
| --- | --- | --- | --- | --- | --- |
| **RefSeq.ID** | **Hugo** | **RefSeq.ID** | **Hugo** | **RefSeq.ID** | **Hugo** |
| NM_000161 | GCH1 | NM_000265 | NCF1 | NM_000051 | ATM |
| NM_000201 | ICAM1 | NM_000304 | PMP22 | NM_000064 | C3 |
| NM_000214 | JAG1 | NM_000396 | CTSK | NM_000118 | ENG |
| NM_000243 | MEFV | NM_000399 | EGR2 | NM_000447 | PSEN2 |
| NM_000245 | MET | NM_000610 | CD44 | NM_000566 | FCGR1A |
| NM_000575 | IL1A | NM_000617 | SLC11A2 | NM_000593 | TAP1 |
| NM_000576 | IL1B | NM_000786 | CYP51A1 | NM_000784 | CYP27A1 |
| NM_000594 | TNF | NM_000859 | HMGCR | NM_000788 | DCK |
| NM_000600 | IL6 | NM_000882 | IL12A | NM_000885 | ITGA4 |
| NM_000675 | ADORA2A | NM_000958 | PTGER4 | NM_000924 | PDE1B |
| NM_000963 | PTGS2 | NM_001001548 | CD36 | NM_000956 | PTGER2 |
| NM_001001435 | CCL4L1 | NM_001002921 | AK3L2 | NM_001002009 | NT5C3 |
| NM_001001437 | CCL3L3 | NM_001004431 | METRNL | NM_001007226 | SPOP |
| NM_001001716 | NFKBIB | NM_001005353 | AK3L1 | NM_001008540 | CXCR4 |
| NM_001004065 | AKAP2 | NM_001025390 | AMPD3 | NM_001015880 | PAPSS2 |
| NM_001004196 | CD200 | NM_001040716 | -1 | NM_001017535 | VDR |
| NM_001007245 | IFRD1 | NM_001042481 | -1 | NM_001017986 | LOC440607 |
| NM_001008490 | KLF6 | NM_001304 | CPD | NM_001018077 | NR3C1 |
| NM_001025300 | RAB12 | NM_001432 | EREG | NM_001025366 | VEGF |
| NM_001037341 | PDE4B | NM_001465 | FYB | NM_001031683 | IFIT3 |
| NM_001042368 | -1 | NM_001482 | GATM | NM_001032283 | TMPO |
| NM_001042483 | -1 | NM_001514 | GTF2B | NM_001037335 | PDIP1beta |
| NM_001066 | TNFRSF1B | NM_001611 | ACP5 | NM_001040078 | -1 |
| NM_001250 | CD40 | NM_001637 | AOAH | NM_001040199 | -1 |
| NM_001381 | DOK1 | NM_001657 | AREG | NM_001165 | BIRC3 |
| NM_001397 | ECE1 | NM_001675 | ATF4 | NM_001350 | DAXX |
| NM_001457 | FLNB | NM_001752 | CAT | NM_001431 | EPB41L2 |
| NM_001511 | CXCL1 | NM_001766 | CD1D | NM_001442 | FABP4 |
| NM_001530 | HIF1A | NM_001772 | CD33 | NM_001491 | GCNT2 |
| NM_001562 | IL18 | NM_001789 | CDC25A | NM_001547 | IFIT2 |
| NM_001565 | CXCL10 | NM_001827 | CKS2 | NM_001552 | IGFBP4 |
| NM_001775 | CD38 | NM_001974 | EMR1 | NM_001710 | CFB |
| NM_001860 | SLC31A2 | NM_002133 | HMOX1 | NM_001839 | CNN3 |
| NM_001924 | GADD45A | NM_002210 | ITGAV | NM_001945 | HBEGF |
| NM_001987 | ETV6 | NM_002254 | KIF3C | NM_001955 | EDN1 |
| NM_001995 | ACSL1 | NM_002258 | KLRB1 | NM_001964 | EGR1 |
| NM_002029 | FPR1 | NM_002337 | LRPAP1 | NM_001993 | F3 |
| NM_002089 | CXCL2 | NM_002348 | LY9 | NM_002024 | FMR1 |
| NM_002110 | HCK | NM_002349 | LY75 | NM_002037 | FYN |
| NM_002187 | IL12B | NM_002358 | MAD2L1 | NM_002068 | GNA15 |
| NM_002192 | INHBA | NM_002359 | MAFG | NM_002114 | HIVEP1 |
| NM_002198 | IRF1 | NM_002543 | OLR1 | NM_002163 | IRF8 |
| NM_002205 | ITGA5 | NM_002641 | PIGA | NM_002182 | IL1RAP |
| NM_002228 | JUN | NM_002649 | PIK3CG | NM_002185 | IL7R |
| NM_002231 | CD82 | NM_002659 | PLAUR | NM_002199 | IRF2 |
| NM_002502 | NFKB2 | NM_002704 | PPBP | NM_002201 | ISG20 |
| NM_002575 | SERPINB2 | NM_002872 | RAC2 | NM_002221 | ITPKB |
| NM_002657 | PLAGL2 | NM_002886 | RAP2B | NM_002222 | ITPR1 |
| NM_002664 | PLEK | NM_002990 | CCL22 | NM_002229 | JUNB |
| NM_002843 | PTPRJ | NM_003037 | SLAMF1 | NM_002357 | MXD1 |
| NM_002852 | PTX3 | NM_003177 | SYK | NM_002397 | MEF2C |
| NM_002856 | PVRL2 | NM_003272 | GPR137B | NM_002436 | MPP1 |
| NM_002894 | RBBP8 | NM_003566 | EEA1 | NM_002462 | MX1 |
| NM_002922 | RGS1 | NM_003677 | DENR | NM_002501 | NFIX |
| NM_002982 | CCL2 | NM_003692 | TMEFF1 | NM_002514 | NOV |
| NM_002983 | CCL3 | NM_003749 | IRS2 | NM_002535 | OAS2 |
| NM_002984 | CCL4 | NM_003983 | SLC7A6 | NM_002573 | PAFAH1B3 |
| NM_002999 | SDC4 | NM_003998 | NFKB1 | NM_002648 | PIM1 |
| NM_003033 | ST3GAL1 | NM_004171 | SLC1A2 | NM_002714 | PPP1R10 |
| NM_003044 | SLC6A12 | NM_004181 | UCHL1 | NM_002729 | HHEX |
| NM_003088 | FSCN1 | NM_004226 | STK17B | NM_002755 | MAP2K1 |
| NM_003264 | TLR2 | NM_004280 | EEF1E1 | NM_002775 | HTRA1 |
| NM_003326 | TNFSF4 | NM_004287 | GOSR2 | NM_002800 | PSMB9 |
| NM_003628 | PKP4 | NM_004337 | C8orf1 | NM_002828 | PTPN2 |
| NM_003745 | SOCS1 | NM_004613 | TGM2 | NM_002901 | RCN1 |
| NM_003811 | TNFSF9 | NM_004741 | NOLC1 | NM_002908 | REL |
| NM_003879 | CFLAR | NM_004938 | DAPK1 | NM_003064 | SLPI |
| NM_003896 | ST3GAL5 | NM_004994 | MMP9 | NM_003105 | SORL1 |
| NM_003955 | SOCS3 | NM_004995 | MMP14 | NM_003115 | UAP1 |
| NM_003956 | CH25H | NM_004996 | ABCC1 | NM_003151 | STAT4 |
| NM_003965 | CCRL2 | NM_004998 | MYO1E | NM_003165 | STXBP1 |
| NM_004040 | RHOB | NM_005047 | PSMD5 | NM_003544 | HIST1H4B |
| NM_004049 | BCL2A1 | NM_005067 | SIAH2 | NM_003581 | NCK2 |
| NM_004102 | FABP3 | NM_005143 | HP | NM_003596 | TPST1 |
| NM_004155 | SERPINB9 | NM_005335 | HCLS1 | NM_003618 | MAP4K3 |
| NM_004180 | TANK | NM_005739 | RASGRP1 | NM_003633 | ENC1 |
| NM_004347 | CASP5 | NM_005786 | SDCCAG33 | NM_003655 | CBX4 |
| NM_004354 | CCNG2 | NM_005923 | MAP3K5 | NM_003733 | OASL |
| NM_004417 | DUSP1 | NM_005955 | MTF1 | NM_003810 | TNFSF10 |
| NM_004556 | NFKBIE | NM_006018 | GPR109B | NM_003821 | RIPK2 |
| NM_004759 | MAPKAPK2 | NM_006118 | HAX1 | NM_004064 | CDKN1B |
| NM_004878 | PTGES | NM_006209 | ENPP2 | NM_004120 | GBP2 |
| NM_005168 | RND3 | NM_006281 | STK3 | NM_004240 | TRIP10 |
| NM_005191 | CD80 | NM_006285 | TESK1 | NM_004242 | HMGN3 |
| NM_005239 | ETS2 | NM_006291 | TNFAIP2 | NM_004251 | RAB9A |
| NM_005252 | FOS | NM_006392 | NOL5A | NM_004272 | HOMER1 |
| NM_005414 | SKIL | NM_006474 | PDPN | NM_004310 | RHOH |
| NM_005415 | SLC20A1 | NM_006522 | WNT6 | NM_004345 | CAMP |
| NM_005417 | SRC | NM_006748 | SLA | NM_004348 | RUNX2 |
| NM_005534 | IFNGR2 | NM_006750 | SNTB2 | NM_004566 | PFKFB3 |
| NM_005565 | LCP2 | NM_006763 | BTG2 | NM_004573 | PLCB2 |
| NM_005658 | TRAF1 | NM_006770 | MARCO | NM_004595 | SMS |
| NM_005755 | EBI3 | NM_006806 | BTG3 | NM_004738 | VAPB |
| NM_006058 | TNIP1 | NM_006875 | PIM2 | NM_004789 | LHX2 |
| NM_006079 | CITED2 | NM_007034 | DNAJB4 | NM_004833 | AIM2 |
| NM_006290 | TNFAIP3 | NM_007046 | EMILIN1 | NM_004906 | WTAP |
| NM_006505 | PVR | NM_007085 | FSTL1 | NM_004926 | ZFP36L1 |
| NM_006506 | RASA2 | NM_012219 | MRAS | NM_004960 | FUS |
| NM_006577 | B3GNT1 | NM_012223 | MYO1B | NM_005101 | G1P2 |
| NM_006622 | PLK2 | NM_012248 | SEPHS2 | NM_005128 | DOPEY2 |
| NM_006795 | EHD1 | NM_012252 | TFEC | NM_005240 | ETV3 |
| NM_006834 | RAB32 | NM_012329 | MMD | NM_005254 | GABPB2 |
| NM_007203 | AKAP2 | NM_013309 | SLC30A4 | NM_005391 | PDK3 |
| NM_007350 | PHLDA1 | NM_013411 | AK2 | NM_005400 | PRKCE |
| NM_012081 | ELL2 | NM_013416 | NCF4 | NM_005409 | CXCL11 |
| NM_012323 | MAFF | NM_014372 | RNF11 | NM_005428 | VAV1 |
| NM_012334 | MYO10 | NM_014398 | LAMP3 | NM_005465 | AKT3 |
| NM_013314 | BLNK | NM_014489 | MGC799 | NM_005582 | CD180 |
| NM_013322 | SNX10 | NM_014585 | SLC40A1 | NM_005744 | ARIH1 |
| NM_013390 | TMEM2 | NM_015122 | FCHO1 | NM_005863 | NET1 |
| NM_014002 | IKBKE | NM_015259 | ICOSLG | NM_005895 | GOLGA3 |
| NM_014143 | CD274 | NM_015359 | SLC39A14 | NM_005900 | SMAD1 |
| NM_014358 | CLEC4E | NM_015577 | RAI14 | NM_005935 | AFF1 |
| NM_014504 | RABGEF1 | NM_015641 | TES | NM_005957 | MTHFR |
| NM_015055 | KIAA0640 | NM_015679 | TRUB2 | NM_005966 | NAB1 |
| NM_015278 | SASH1 | NM_016227 | C1orf9 | NM_006005 | WFS1 |
| NM_015675 | GADD45B | NM_016395 | PTPLAD1 | NM_006186 | NR4A2 |
| NM_015900 | PLA1A | NM_016582 | SLC15A3 | NM_006243 | PPP2R5A |
| NM_016562 | TLR7 | NM_016607 | ARMCX3 | NM_006273 | CCL7 |
| NM_016612 | SLC25A37 | NM_017447 | C21orf91 | NM_006309 | LRRFIP2 |
| NM_017817 | RAB20 | NM_017964 | SLC30A6 | NM_006343 | MERTK |
| NM_018266 | TMEM39A | NM_018010 | IFT57 | NM_006378 | SEMA4D |
| NM_018351 | FGD6 | NM_018509 | LRRC59 | NM_006417 | IFI44 |
| NM_019555 | ARHGEF3 | NM_018643 | TREM1 | NM_006457 | PDLIM5 |
| NM_020370 | GPR84 | NM_018677 | ACSS2 | NM_006480 | RGS14 |
| NM_020529 | NFKBIA | NM_018841 | GNG12 | NM_006509 | RELB |
| NM_021006 | CCL3L1 | NM_018941 | CLN8 | NM_006623 | PHGDH |
| NM_021181 | SLAMF7 | NM_018948 | ERRFI1 | NM_006633 | IQGAP2 |
| NM_022485 | C3orf29 | NM_019023 | KIAA1933 | NM_006682 | FGL2 |
| NM_023009 | MARCKSL1 | NM_019091 | PLEKHA3 | NM_006726 | LRBA |
| NM_030627 | CPEB4 | NM_019112 | ABCA7 | NM_006803 | AP3M2 |
| NM_030640 | DUSP16 | NM_019618 | IL1F9 | NM_006813 | PNRC1 |
| NM_031419 | NFKBIZ | NM_019886 | CHST7 | NM_006889 | CD86 |
| NM_032199 | ARID5B | NM_020132 | AGPAT3 | NM_006989 | RASA4 |
| NM_052862 | RCSD1 | NM_020234 | DTWD1 | NM_007069 | HRASLS3 |
| NM_057178 | RFFL | NM_020313 | CIAPIN1 | NM_007283 | MGLL |
| NM_080927 | DCBLD2 | NM_020432 | PHTF2 | NM_007360 | KLRK1 |
| NM_144650 | ADHFE1 | NM_020754 | MGC138370 | NM_012198 | GCA |
| NM_152309 | PIK3AP1 | NM_020806 | GPHN | NM_012244 | SLC7A8 |
| NM_152594 | SPRED1 | NM_020895 | GRAMD1A | NM_012255 | XRN2 |
| NM_153023 | SPATA13 | NM_021202 | TP53INP2 | NM_012342 | BAMBI |
| NM_153259 | MCOLN2 | NM_021642 | FCGR2A | NM_012388 | PLDN |
| NM_172200 | IL15RA | NM_021908 | ST7 | NM_012395 | PFTK1 |
| NM_173843 | IL1RN | NM_022136 | SAMSN1 | NM_013233 | STK39 |
| NM_174907 | PPP4R2 | NM_022465 | ZNFN1A4 | NM_013254 | TBK1 |
| NM_178562 | TSPAN33 | NM_022566 | MESDC1 | NM_014290 | TDRD7 |
| NM_183380 | DST | NM_022977 | ACSL4 | NM_014395 | DAPP1 |
| NM_198578 | LRRK2 | NM_024324 | CRELD2 | NM_014399 | TSPAN13 |
| NM_198584 | CA13 | NM_024420 | PLA2G4A | NM_014701 | KIAA0256 |
| NM_207007 | CCL4L2 | NM_024430 | PSTPIP2 | NM_014734 | KIAA0247 |
| NM_207585 | IFNAR2 | NM_024640 | YRDC | NM_014828 | C14orf92 |
|  |  | NM_024940 | DOCK5 | NM_014936 | ENPP4 |
|  |  | NM_025079 | ZC3H12A | NM_014961 | RUFY3 |
|  |  | NM_025195 | TRIB1 | NM_014999 | RAB21 |
|  |  | NM_030767 | AKNA | NM_015173 | TBC1D1 |
|  |  | NM_032027 | TM2D1 | NM_015184 | PLCL2 |
|  |  | NM_032194 | BXDC1 | NM_015322 | FEM1B |
|  |  | NM_032351 | MRPL45 | NM_015368 | PANX1 |
|  |  | NM_032564 | DGAT2 | NM_015662 | IFT172 |
|  |  | NM_033514 | LIMS3 | NM_016245 | DHRS8 |
|  |  | NM_078487 | CDKN2B | NM_016320 | NUP98 |
|  |  | NM_080387 | CLEC4D | NM_016507 | CRKRS |
|  |  | NM_080546 | SLC44A1 | NM_016516 | VPS54 |
|  |  | NM_080655 | C9orf30 | NM_016545 | IER5 |
|  |  | NM_138459 | C6orf68 | NM_016584 | IL23A |
|  |  | NM_138714 | NFAT5 | NM_016619 | PLAC8 |
|  |  | NM_139018 | CD300LF | NM_016621 | PHF21A |
|  |  | NM_144726 | DKFZp686M11215 | NM_017414 | USP18 |
|  |  | NM_145206 | VTI1A | NM_017554 | PARP14 |
|  |  | NM_145341 | PDCD4 | NM_017556 | FBLIM1 |
|  |  | NM_152280 | SYT11 | NM_017798 | YTHDF1 |
|  |  | NM_152392 | AHSA2 | NM_018169 | C12orf35 |
|  |  | NM_152396 | METTL6 | NM_018344 | SLC29A3 |
|  |  | NM_171982 | TRIM35 | NM_018360 | CXorf15 |
|  |  | NM_172374 | IL4I1 | NM_018370 | FLJ11259 |
|  |  | NM_173694 | ATP11C | NM_018425 | RP11-548K23.6 |
|  |  | NM_173854 | SLC41A1 | NM_018695 | ERBB2IP |
|  |  | NM_175073 | APTX | NM_018697 | LANCL2 |
|  |  | NM_177551 | GPR109A | NM_018955 | UBB |
|  |  | NM_181523 | PIK3R1 | NM_018993 | RIN2 |
|  |  | NM_181597 | UPP1 | NM_020235 | BBX |
|  |  | NM_181784 | SPRED2 | NM_020244 | CHPT1 |
|  |  | NM_182491 | ZFAND2A | NM_020428 | SLC44A2 |
|  |  | NM_182961 | SYNE1 | NM_020651 | PELI1 |
|  |  | NM_198402 | PTPLB | NM_020698 | TMCC3 |
|  |  | NM_198563 | TMEM110 | NM_020727 | ZNF295 |
|  |  | NM_201433 | GAS7 | NM_020765 | RP5-1126H10.1 |
|  |  | NM_203380 | ACSL5 | NM_021035 | FLJ11277 |
|  |  | NM_212482 | FN1 | NM_021138 | TRAF2 |
|  |  |  |  | NM_021158 | TRIB3 |
|  |  |  |  | NM_021818 | SAV1 |
|  |  |  |  | NM_022168 | IFIH1 |
|  |  |  |  | NM_022337 | RAB38 |
|  |  |  |  | NM_022343 | C9orf19 |
|  |  |  |  | NM_022371 | TOR3A |
|  |  |  |  | NM_022740 | HIPK2 |
|  |  |  |  | NM_022771 | TBC1D15 |
|  |  |  |  | NM_023080 | C8orf33 |
|  |  |  |  | NM_023927 | GRAMD3 |
|  |  |  |  | NM_024098 | FLJ22321 |
|  |  |  |  | NM_024119 | D11lgp2e |
|  |  |  |  | NM_024576 | OGFRL1 |
|  |  |  |  | NM_024613 | PLEKHF2 |
|  |  |  |  | NM_024701 | ASB13 |
|  |  |  |  | NM_024828 | C9orf82 |
|  |  |  |  | NM_030667 | PTPRO |
|  |  |  |  | NM_030751 | TCF8 |
|  |  |  |  | NM_030776 | ZBP1 |
|  |  |  |  | NM_031212 | SLC25A28 |
|  |  |  |  | NM_032047 | B3GNT5 |
|  |  |  |  | NM_032217 | ANKRD17 |
|  |  |  |  | NM_032322 | RNF135 |
|  |  |  |  | NM_032501 | ACSS1 |
|  |  |  |  | NM_032603 | LOXL3 |
|  |  |  |  | NM_032682 | FOXP1 |
|  |  |  |  | NM_032802 | PSL2 |
|  |  |  |  | NM_032810 | ATAD1 |
|  |  |  |  | NM_032813 | TMTC4 |
|  |  |  |  | NM_032833 | PPP1R15B |
|  |  |  |  | NM_033222 | PSIP1 |
|  |  |  |  | NM_033292 | CASP1 |
|  |  |  |  | NM_033338 | CASP7 |
|  |  |  |  | NM_033394 | TANC1 |
|  |  |  |  | NM_033445 | HIST3H2A |
|  |  |  |  | NM_033547 | MGC16733 |
|  |  |  |  | NM_052868 | IGSF8 |
|  |  |  |  | NM_053056 | CCND1 |
|  |  |  |  | NM_080657 | RSAD2 |
|  |  |  |  | NM_080794 | MRPL39 |
|  |  |  |  | NM_130439 | MXI1 |
|  |  |  |  | NM_133436 | ASNS |
|  |  |  |  | NM_133496 | SLC30A7 |
|  |  |  |  | NM_138335 | GNPDA2 |
|  |  |  |  | NM_138426 | GLCCI1 |
|  |  |  |  | NM_138931 | BCL6 |
|  |  |  |  | NM_145034 | TOR1AIP2 |
|  |  |  |  | NM_145041 | TMEM106A |
|  |  |  |  | NM_145071 | CISH |
|  |  |  |  | NM_147223 | NCOA1 |
|  |  |  |  | NM_152270 | SLFN8/9 |
|  |  |  |  | NM_152641 | ARID2 |
|  |  |  |  | NM_153207 | AEBP2 |
|  |  |  |  | NM_172174 | IL15 |
|  |  |  |  | NM_173042 | IL18BP |
|  |  |  |  | NM_173074 | PIGF |
|  |  |  |  | NM_173174 | PTK2B |
|  |  |  |  | NM_175623 | RAB3IP |
|  |  |  |  | NM_178155 | FUT8 |
|  |  |  |  | NM_181755 | HSD11B1 |
|  |  |  |  | NM_181795 | PKIB |
|  |  |  |  | NM_181805 | PKIG |
|  |  |  |  | NM_182488 | USP12 |
|  |  |  |  | NM_182526 | FLJ33387 |
|  |  |  |  | NM_182926 | KTN1 |
|  |  |  |  | NM_182943 | PLOD2 |
|  |  |  |  | NM_183353 | RNF12 |
|  |  |  |  | NM_194449 | PHLPP |
|  |  |  |  | NM_197966 | BID |
|  |  |  |  | NM_198040 | PHC2 |
|  |  |  |  | NM_198460 | GBP6 |
|  |  |  |  | NM_199072 | MDFIC |
|  |  |  |  | NM_199344 | SFT2D2 |
