# Supplementary material for: Functional Comparison of Innate Immune Signaling Pathways in Primates
Source: PLoS Genet. 2010 Dec 16;6(12):e1001249. doi: 10.1371/journal.pgen.1001249 (PMC3002988; doi:10.1371/journal.pgen.1001249)
Supplement: Table S5 — KEGG pathways enrichment analyzes for the 335 genes that responded to LPS only in humans. (0.05 MB DOC) [file pgen.1001249.s021.doc]

| **KEGG pathways** | | | | | |
| --- | --- | --- | --- | --- | --- |
| **Subcategory** | **Subcategory alternative name** | **expected** | **observed** | **P-value (raw)erĀ P-value (FDR)( 00Pathways in cancer0µ0µRenal cell carcinoma 00Endometrial** | **P-value (FDR)( 00Pathways in cancer** |
| Prostate cancer | 5215 | 1.62461 | 7 | 0.001048 | 0.0504887 |
| Insulin signaling pathway | 4910 | 2.1804 | 8 | 0.001329 | 0.0504887 |
| Chronic myeloid leukemia | 5220 | 1.4536 | 6 | 0.003018 | 0.0617409 |
| B cell receptor signaling pathway | 4662 | 1.47497 | 6 | 0.00325 | 0.0617409 |
| Nitrogen metabolism | 910 | 0.342023 | 3 | 0.004313 | 0.0655581 |
| Apoptosis | 4210 | 1.62461 | 6 | 0.005263 | 0.0666692 |
| Pancreatic cancer | 5212 | 1.26121 | 5 | 0.008007 | 0.0869284 |
| mTOR signaling pathway | 4150 | 0.89781 | 4 | 0.011716 | 0.111298 |
| Glycine, serine and threonine metabolism | 260 | 0.534411 | 3 | 0.015429 | 0.111462 |
| Non-small cell lung cancer | 5223 | 0.983316 | 4 | 0.016017 | 0.111462 |
| Small cell lung cancer | 5222 | 1.49635 | 5 | 0.016133 | 0.111462 |
| Adipocytokine signaling pathway | 4920 | 1.04745 | 4 | 0.01982 | 0.125525 |
| Melanoma | 5218 | 1.0902 | 4 | 0.02264 | 0.127617 |
| MAPK signaling pathway | 4010 | 4.27529 | 9 | 0.025561 | 0.127617 |
| Glioma | 5214 | 1.13295 | 4 | 0.025694 | 0.127617 |
| Natural killer cell mediated cytotoxicity | 4650 | 2.30865 | 6 | 0.026867 | 0.127617 |
| Acute myeloid leukemia | 5221 | 1.1757 | 4 | 0.028986 | 0.129583 |
| Type II diabetes mellitus | 4930 | 0.705422 | 3 | 0.032405 | 0.130708 |
| Toll-like receptor signaling pathway | 4620 | 1.79562 | 5 | 0.032677 | 0.130708 |
| Pathways in cancer | 5200 | 5.30136 | 10 | 0.036918 | 0.140288 |
| Renal cell carcinoma | 5211 | 1.34672 | 4 | 0.044574 | 0.157725 |
| Chagas disease | 5142 | 1.96663 | 5 | 0.045657 | 0.157725 |
