# Supplementary material for: Functional Comparison of Innate Immune Signaling Pathways in Primates
Source: PLoS Genet. 2010 Dec 16;6(12):e1001249. doi: 10.1371/journal.pgen.1001249 (PMC3002988; doi:10.1371/journal.pgen.1001249)
Supplement: Table S6 — KEGG pathways enrichment analyzes for the 273 genes that responded to LPS only in chimpanzees. (0.03 MB DOC) [file pgen.1001249.s022.doc]

| **KEGG pathways** | | | | | |
| --- | --- | --- | --- | --- | --- |
| **Subcategory** | **Subcategory alternative name** | **expected** | **observed** | **P-value (raw)erĀ P-value (FDR)(  Ȁ0Pathways in cancer ̀µ0µRenal cell carcinoma00Endometrial** | **P-value (FDR)(  Ȁ0Pathways in cancer** |
| Phagosome | 4145 | 3.32847 | 10 | 0.00158945 | 0.0905987 |
| Systemic lupus erythematosus | 5322 | 2.42753 | 7 | 0.0101117 | >0.2 |
| Leishmaniasis | 5140 | 1.55162 | 5 | 0.0185748 | >0.2 |
| Selenoamino acid metabolism | 450 | 0.5756 | 3 | 0.0187137 | >0.2 |
| Cell adhesion molecules (CAMs) | 4514 | 2.3024 | 6 | 0.0265854 | >0.2 |
| Pathogenic Escherichia coli infection | 5130 | 1.2513 | 4 | 0.0352754 | >0.2 |
