# Supplementary material for: Functional Comparison of Innate Immune Signaling Pathways in Primates
Source: PLoS Genet. 2010 Dec 16;6(12):e1001249. doi: 10.1371/journal.pgen.1001249 (PMC3002988; doi:10.1371/journal.pgen.1001249)
Supplement: Table S7 — KEGG pathways enrichment analyzes for the 393 genes that responded to LPS only in rhesus macaques. (0.03 MB DOC) [file pgen.1001249.s023.doc]

| **KEGG pathways** | | | | | |
| --- | --- | --- | --- | --- | --- |
| **Subcategory** | **Subcategory alternative name** | **expected** | **observed** | ***P-value* (raw)** | ***P-value* (FDR)** |
| Synthesis and degradation of ketone bodies | 72 | 0.209854 | 2 | 0.0169546 | >0.2 |
| Long-term depression | 4730 | 1.52894 | 5 | 0.017378 | >0.2 |
| Small cell lung cancer | 5222 | 2.09854 | 6 | 0.0175442 | >0.2 |
| Cell cycle | 4110 | 3.47758 | 8 | 0.0219816 | >0.2 |
| Vascular smooth muscle contraction | 4270 | 2.45829 | 6 | 0.0351344 | >0.2 |
| Fc epsilon RI signaling pathway | 4664 | 1.85871 | 5 | 0.0369747 | >0.2 |
| Non-homologous end-joining | 3450 | 0.35975 | 2 | 0.0483323 | >0.2 |
