# Supplementary material for: Functional Comparison of Innate Immune Signaling Pathways in Primates
Source: PLoS Genet. 2010 Dec 16;6(12):e1001249. doi: 10.1371/journal.pgen.1001249 (PMC3002988; doi:10.1371/journal.pgen.1001249)
Supplement: Table S8 — Functional interaction scores between the set of 335 genes that respond to LPS exclusively in humans. Only interactions with a score higher than 0.4 (the default cutoff in STRING) are shown. (0.17 MB DOC) [file pgen.1001249.s024.doc]

| **node1** | **node2** | **Confidence score** |
| --- | --- | --- |
| AGPAT4 | AGPAT6 | 0.874 |
| C1orf59 | GEMIN5 | 0.508 |
| SP1 | HCK | 0.861 |
| ZNF652 | CBFA2T3 | 0.974 |
| FABP3 | AGPAT6 | 0.647 |
| LRPAP1 | NOL6 | 0.457 |
| ECE1 | RELA | 0.899 |
| SLC7A1 | SLC38A2 | 0.498 |
| ACVR2A | FSTL3 | 0.469 |
| DTNBP1 | AKT1 | 0.769 |
| PHGDH | GLUD1 | 0.686 |
| PTPN22 | GIMAP4 | 0.484 |
| MTMR12 | SET | 0.706 |
| GRPR | SDSL | 0.466 |
| SLC38A2 | SLC1A4 | 0.637 |
| GRB2 | PTK2B | 0.991 |
| ECHDC2 | EIF2AK4 | 0.469 |
| TRAF3 | RELA | 0.507 |
| TGFBR1 | PIK3R1 | 0.625 |
| HDAC11 | HDAC4 | 0.926 |
| PHGDH | SLC1A4 | 0.716 |
| PLK1 | C20orf19 | 0.899 |
| CREB3L2 | RND1 | 0.553 |
| ECE1 | E2F2 | 0.46 |
| HOXA9 | AFF1 | 0.454 |
| LRPAP1 | AKT1 | 0.791 |
| TAP1 | SLC38A2 | 0.48 |
| SLC29A1 | TAP1 | 0.623 |
| OLR1 | HSPA2 | 0.512 |
| PTK2B | PIK3R1 | 0.773 |
| VTI1A | GOSR2 | 0.707 |
| OPRL1 | OLR1 | 0.456 |
| POP1 | RPP30 | 0.771 |
| ACACB | CAB39 | 0.899 |
| FABP3 | PPARA | 0.595 |
| MTMR12 | PIK3R1 | 0.402 |
| CD1D | KLRB1 | 0.88 |
| LRPAP1 | RASGRP3 | 0.653 |
| GRB2 | NEU3 | 0.703 |
| KCNJ11 | GLUD1 | 0.802 |
| DAXX | ACVR2A | 0.631 |
| HIF1A | AKT1 | 0.997 |
| PPARA | ABHD4 | 0.42 |
| IRS2 | GRB2 | 0.998 |
| GLUL | TAP1 | 0.4 |
| KCNE1L | NXT2 | 0.56 |
| SPEN | PIK3R1 | 0.433 |
| GLUL | GLUD1 | 0.975 |
| GLUD1 | SDS | 0.609 |
| GCN1L1 | EIF2AK4 | 0.981 |
| SP1 | KLF10 | 0.546 |
| RASGRP3 | RASGRP4 | 0.817 |
| GRB2 | FGFR1 | 0.995 |
| ECE1 | KRT222P | 0.899 |
| RNF38 | GNE | 0.563 |
| GRPR | SP1 | 0.899 |
| SP1 | RELA | 0.971 |
| HIF1A | HDAC4 | 0.43 |
| IRS2 | AKT1 | 0.987 |
| HIF1A | RELA | 0.626 |
| MLLT6 | AFF1 | 0.86 |
| SMAP1L | AKT1 | 0.405 |
| RUNX2 | RELA | 0.434 |
| TAP1 | STEAP3 | 0.441 |
| KRT222P | RELA | 0.899 |
| SP1 | FGFR1 | 0.639 |
| GRB2 | AKT1 | 0.992 |
| HIF1A | SP1 | 0.97 |
| HIF1A | PPARA | 0.857 |
| PPP1CC | DONSON | 0.524 |
| GRPR | RELA | 0.899 |
| COL18A1 | HPSE | 0.411 |
| RUNX2 | HDAC4 | 0.976 |
| PTPN22 | PXK | 0.731 |
| OLR1 | PPARA | 0.673 |
| PTK2B | HCK | 0.916 |
| TGFBR1 | SPTBN1 | 0.899 |
| LRPAP1 | PRKAR1A | 0.409 |
| PTPN22 | GRB2 | 0.96 |
| KIR2DL1 | KLRB1 | 0.806 |
| OLR1 | AKT1 | 0.439 |
| GNE | NRGN | 0.441 |
| IPO4 | POU2F3 | 0.48 |
| PPP1CC | PLK1 | 0.899 |
| SLC7A1 | SLC7A7 | 0.41 |
| SLC6A12 | TAP1 | 0.591 |
| IRS2 | PIK3R1 | 0.999 |
| GRB2 | P2RX7 | 0.414 |
| ACACB | DGAT2 | 0.605 |
| COL18A1 | RELA | 0.899 |
| HMGA2 | NOL6 | 0.424 |
| IFNGR1 | PIK3R1 | 0.899 |
| KRT222P | SMARCA2 | 0.994 |
| PTPN22 | ABHD4 | 0.527 |
| SLC6A12 | SLC38A2 | 0.619 |
| LRPAP1 | RAB35 | 0.669 |
| DTNBP1 | ST3GAL1 | 0.441 |
| MLLT6 | ELL | 0.462 |
| SPEN | LIME1 | 0.425 |
| SLC7A1 | SLC29A1 | 0.436 |
| DAXX | MX1 | 0.629 |
| PIK3R1 | HCK | 0.565 |
| PIK3R1 | AKT1 | 0.997 |
| MVD | PWP2 | 0.538 |
| PRKAR1A | AKT1 | 0.658 |
| FABP3 | MTG1 | 0.48 |
| LRPAP1 | RASGRP4 | 0.457 |
| RELA | PPARA | 0.756 |
| GRPR | GLUD1 | 0.427 |
| TAP1 | SLC7A7 | 0.679 |
| ST3GAL1 | ST3GAL4 | 0.864 |
| FGL2 | PROC | 0.604 |
| ABL2 | ACO1 | 0.406 |
| GLUL | SDS | 0.696 |
| TAP1 | ACO1 | 0.681 |
| PRKAR1A | GRB2 | 0.78 |
| TAP1 | SLC15A3 | 0.452 |
| ACAA1 | TYSND1 | 0.55 |
| KRT13 | WNT6 | 0.408 |
| ABL2 | CASP10 | 0.461 |
| UBE2G1 | PROC | 0.475 |
| AKT1 | HCK | 0.597 |
| PHGDH | GLUL | 0.499 |
| KCTD12 | GLUD1 | 0.617 |
| TAP1 | HIF1A | 0.793 |
| PTK2B | ARHGAP26 | 0.517 |
| ABL2 | AFF1 | 0.462 |
| MLLT6 | PCQAP | 0.433 |
| GRB2 | HCK | 0.913 |
| SLC7A1 | FAM46A | 0.489 |
| CLEC2D | KLRB1 | 0.973 |
| BTG2 | LYL1 | 0.486 |
| KCNJ11 | SP1 | 0.403 |
| SP1 | KLF4 | 0.648 |
| KCNJ11 | GLIS3 | 0.474 |
| CTPS | POLR1A | 0.899 |
| PHGDH | SDS | 0.73 |
| GRB2 | PIK3R1 | 0.999 |
| GRPR | COL18A1 | 0.899 |
| MLLT6 | ARHGAP26 | 0.633 |
| HMGA2 | RELA | 0.698 |
| TAP1 | SLC37A3 | 0.593 |
| RELA | AKT1 | 0.972 |
| RELA | PIK3R1 | 0.927 |
| CTPS | AMT | 0.666 |
| GEMIN4 | GEMIN5 | 0.997 |
| TAP1 | DTNBP1 | 0.405 |
| KCNE1L | KCNJ11 | 0.417 |
| AFF1 | ELL | 0.776 |
| TAP1 | SLC1A4 | 0.511 |
| ECE1 | C20orf108 | 0.424 |
| LYL1 | HCK | 0.4 |
| TRAF3 | FGL2 | 0.48 |
| RASGRP3 | RAP2B | 0.681 |
| HIF1A | PIK3R1 | 0.496 |
| RUNX2 | GLIS3 | 0.454 |
| TGFBR1 | RUNX2 | 0.916 |
| CD86 | KLRB1 | 0.414 |
| GNE | UXS1 | 0.571 |
| MX1 | ST3GAL4 | 0.734 |
| SLC19A1 | TCN2 | 0.698 |
| GRPR | PTK2B | 0.45 |
| NINJ1 | BTG2 | 0.538 |
| FLOT1 | PTK2B | 0.52 |
| ACACB | PPARA | 0.463 |
| CTDSPL | HDAC4 | 0.585 |
| PDCD11 | RELA | 0.801 |
| LRP12 | PROC | 0.414 |
| CARD14 | RELA | 0.512 |
| E2F2 | CDK3 | 0.855 |
| AGPAT4 | ING2 | 0.675 |
| COL18A1 | SLC19A1 | 0.499 |
| LRPAP1 | RND1 | 0.491 |
| CD1D | CD86 | 0.726 |
| SET | PPP1CC | 0.477 |
| ABL2 | HCK | 0.633 |
| RUNX2 | FGFR1 | 0.982 |
| DAXX | CASP10 | 0.899 |
| SLC43A2 | SLC38A2 | 0.627 |
| RUNX2 | CBFA2T3 | 0.597 |
| UXS1 | AKT1 | 0.714 |
| PPP1CC | AKT1 | 0.835 |
| E2F2 | SP1 | 0.697 |
| FGFR1 | PIK3R1 | 0.943 |
| GRB2 | CD86 | 0.698 |
| LRPAP1 | RAP2B | 0.746 |
| ACO1 | UBE2R2 | 0.577 |
| CD86 | RELA | 0.626 |
| AKTIP | AKT1 | 0.861 |
| COL18A1 | SP1 | 0.899 |
| GRB2 | LIME1 | 0.623 |
| NOLC1 | POLR1A | 0.853 |
