# Supplementary material for: Functional Comparison of Innate Immune Signaling Pathways in Primates
Source: PLoS Genet. 2010 Dec 16;6(12):e1001249. doi: 10.1371/journal.pgen.1001249 (PMC3002988; doi:10.1371/journal.pgen.1001249)
Supplement: Table S9 — Sources of information used to support evidence of a functional interaction between genes that responded to LPS only in humans. (0.13 MB DOC) [file pgen.1001249.s025.doc]

| **node1** | **node2** | **co-occurrence^1^** | **Homology^2^** | **Experimental^3^** | **Knowledge^4^** | **Text-mining^5^** | **Overall-score** |
| --- | --- | --- | --- | --- | --- | --- | --- |
| AGPAT4 | AGPAT6 | 0 | 0 | 0 | 0.8 | 0.412 | 0.874 |
| RELA | PPARA | 0 | 0 | 0.633 | 0 | 0.379 | 0.756 |
| SP1 | HCK | 0 | 0 | 0 | 0 | 0.861 | 0.861 |
| ST3GAL1 | ST3GAL4 | 0 | 0.627 | 0 | 0.8 | 0.878 | 0.864 |
| ZNF652 | CBFA2T3 | 0 | 0 | 0.626 | 0 | 0.935 | 0.974 |
| ECE1 | RELA | 0 | 0 | 0 | 0.9 | 0 | 0.899 |
| PRKAR1A | GRB2 | 0 | 0 | 0.626 | 0 | 0.45 | 0.78 |
| DTNBP1 | AKT1 | 0 | 0 | 0 | 0 | 0.769 | 0.769 |
| GRB2 | PTK2B | 0 | 0 | 0.852 | 0 | 0.946 | 0.991 |
| HDAC11 | HDAC4 | 0.485 | 0.637 | 0 | 0.9 | 0.371 | 0.926 |
| PHGDH | SLC1A4 | 0 | 0 | 0 | 0 | 0.717 | 0.716 |
| PLK1 | C20orf19 | 0 | 0 | 0 | 0.9 | 0 | 0.899 |
| TAP1 | HIF1A | 0 | 0 | 0 | 0 | 0.793 | 0.793 |
| LRPAP1 | AKT1 | 0 | 0 | 0 | 0 | 0.791 | 0.791 |
| VTI1A | GOSR2 | 0 | 0 | 0 | 0 | 0.707 | 0.707 |
| PTK2B | PIK3R1 | 0 | 0 | 0.633 | 0 | 0.422 | 0.773 |
| POP1 | RPP30 | 0 | 0 | 0.632 | 0 | 0.417 | 0.771 |
| GRB2 | HCK | 0 | 0.779 | 0 | 0.9 | 0.622 | 0.913 |
| ACACB | CAB39 | 0 | 0 | 0 | 0.9 | 0 | 0.899 |
| CLEC2D | KLRB1 | 0 | 0 | 0.633 | 0 | 0.932 | 0.973 |
| CD1D | KLRB1 | 0 | 0 | 0 | 0 | 0.88 | 0.88 |
| GRB2 | NEU3 | 0 | 0 | 0.633 | 0 | 0.242 | 0.703 |
| CTPS | POLR1A | 0 | 0 | 0 | 0.9 | 0.016 | 0.899 |
| KCNJ11 | GLUD1 | 0 | 0 | 0 | 0 | 0.802 | 0.802 |
| PHGDH | SDS | 0 | 0 | 0 | 0 | 0.73 | 0.73 |
| HIF1A | AKT1 | 0 | 0 | 0 | 0.9 | 0.974 | 0.997 |
| GRB2 | PIK3R1 | 0 | 0.653 | 0.99 | 0.9 | 0.976 | 0.999 |
| IRS2 | GRB2 | 0 | 0 | 0.633 | 0.9 | 0.959 | 0.998 |
| GRPR | COL18A1 | 0 | 0 | 0 | 0.9 | 0 | 0.899 |
| GLUL | GLUD1 | 0 | 0 | 0 | 0.9 | 0.768 | 0.975 |
| GCN1L1 | EIF2AK4 | 0 | 0 | 0 | 0 | 0.981 | 0.981 |
| RELA | AKT1 | 0 | 0 | 0 | 0.8 | 0.868 | 0.972 |
| RELA | PIK3R1 | 0 | 0 | 0 | 0.9 | 0.322 | 0.927 |
| RASGRP3 | RASGRP4 | 0 | 0.899 | 0 | 0.8 | 0.895 | 0.817 |
| GEMIN4 | GEMIN5 | 0 | 0 | 0.526 | 0.9 | 0.956 | 0.997 |
| GRB2 | FGFR1 | 0 | 0 | 0.633 | 0.8 | 0.951 | 0.995 |
| ECE1 | KRT222P | 0 | 0 | 0 | 0.9 | 0 | 0.899 |
| GRPR | SP1 | 0 | 0 | 0 | 0.9 | 0 | 0.899 |
| AFF1 | ELL | 0 | 0 | 0 | 0 | 0.776 | 0.776 |
| SP1 | RELA | 0 | 0 | 0.626 | 0.9 | 0.341 | 0.971 |
| IRS2 | AKT1 | 0 | 0 | 0 | 0.9 | 0.885 | 0.987 |
| MLLT6 | AFF1 | 0 | 0 | 0 | 0 | 0.86 | 0.86 |
| TGFBR1 | RUNX2 | 0 | 0 | 0 | 0 | 0.916 | 0.916 |
| KRT222P | RELA | 0 | 0 | 0 | 0.9 | 0 | 0.899 |
| GRB2 | AKT1 | 0 | 0 | 0 | 0.9 | 0.93 | 0.992 |
| HIF1A | SP1 | 0 | 0 | 0.626 | 0.9 | 0.314 | 0.97 |
| HIF1A | PPARA | 0 | 0 | 0 | 0 | 0.857 | 0.857 |
| GRPR | RELA | 0 | 0 | 0 | 0.9 | 0 | 0.899 |
| RUNX2 | HDAC4 | 0 | 0 | 0.633 | 0 | 0.938 | 0.976 |
| MX1 | ST3GAL4 | 0 | 0 | 0 | 0 | 0.734 | 0.734 |
| PTPN22 | PXK | 0 | 0 | 0 | 0 | 0.731 | 0.731 |
| TGFBR1 | SPTBN1 | 0 | 0 | 0 | 0.9 | 0 | 0.899 |
| PTK2B | HCK | 0 | 0.784 | 0 | 0.9 | 0.777 | 0.916 |
| PTPN22 | GRB2 | 0 | 0 | 0.633 | 0 | 0.899 | 0.96 |
| KIR2DL1 | KLRB1 | 0 | 0 | 0 | 0 | 0.806 | 0.806 |
| PPP1CC | PLK1 | 0 | 0 | 0 | 0.9 | 0.024 | 0.899 |
| IRS2 | PIK3R1 | 0 | 0 | 0.979 | 0.9 | 0.946 | 0.999 |
| PDCD11 | RELA | 0 | 0 | 0.633 | 0 | 0.493 | 0.801 |
| COL18A1 | RELA | 0 | 0 | 0 | 0.9 | 0 | 0.899 |
| E2F2 | CDK3 | 0 | 0 | 0.626 | 0 | 0.636 | 0.855 |
| IFNGR1 | PIK3R1 | 0 | 0 | 0 | 0.9 | 0 | 0.899 |
| KRT222P | SMARCA2 | 0 | 0 | 0.61 | 0.9 | 0.873 | 0.994 |
| CD1D | CD86 | 0 | 0 | 0 | 0 | 0.726 | 0.726 |
| RUNX2 | FGFR1 | 0 | 0 | 0 | 0 | 0.982 | 0.982 |
| DAXX | CASP10 | 0 | 0 | 0 | 0.9 | 0 | 0.899 |
| PIK3R1 | AKT1 | 0 | 0 | 0.633 | 0.9 | 0.944 | 0.997 |
| UXS1 | AKT1 | 0 | 0 | 0.633 | 0 | 0.27 | 0.714 |
| PPP1CC | AKT1 | 0 | 0 | 0 | 0.8 | 0.23 | 0.835 |
| FGFR1 | PIK3R1 | 0 | 0 | 0.632 | 0.8 | 0.326 | 0.943 |
| LRPAP1 | RAP2B | 0 | 0 | 0 | 0 | 0.747 | 0.746 |
| AKTIP | AKT1 | 0 | 0 | 0.633 | 0 | 0.647 | 0.861 |
| COL18A1 | SP1 | 0 | 0 | 0 | 0.9 | 0.019 | 0.899 |
| NOLC1 | POLR1A | 0 | 0 | 0.633 | 0 | 0.625 | 0.853 |

^1^ refers to evidence of co-occurrence-patterns of multiple genes across several genomes; ^2^refers to evidence of homology between genes; ^3^refers to experimental evidence; ^4^ refers to previous, well established, knowledge; ^5^ refers to evidence provided by a systematic data-mining of PubMed literature.
