# Supplementary material for: Functional Comparison of Innate Immune Signaling Pathways in Primates
Source: PLoS Genet. 2010 Dec 16;6(12):e1001249. doi: 10.1371/journal.pgen.1001249 (PMC3002988; doi:10.1371/journal.pgen.1001249)
Supplement: Table S13 — Details on the samples used in this study (0.04 MB DOC) [file pgen.1001249.s029.doc]

| **Study Code** | **Species** | **ID** | **Sex** |
| --- | --- | --- | --- |
| H1 | *Homo sapiens* | NA | male |
| H2 | *Homo sapiens* | NA | male |
| H3 | *Homo sapiens* | NA | male |
| H4 | *Homo sapiens* | NA | female |
| H5 | *Homo sapiens* | NA | female |
| H6 | *Homo sapiens* | NA | female |
| C1 | *Pan troglodytes* | Jarred | male |
| C2 | *Pan troglodytes* | Travis | male |
| C3 | *Pan troglodytes* | David | male |
| C4 | *Pan troglodytes* | Sabrina | female |
| C5 | *Pan troglodytes* | Sara | female |
| C6 | *Pan troglodytes* | Rita | female |
| R1 | *Rhesus macaques* | RNF10 | male |
| R2 | *Rhesus macaques* | FH21 | male |
| R3 | *Rhesus macaques* | REO9 | male |
| R4 | *Rhesus macaques* | RJR10 | female |
| R5 | *Rhesus macaques* | RTG10 | female |
| R6 | *Rhesus macaques* | RKS10 | female |

NA: Not available. All human samples were anonymous.
