# Supplementary material for: Functional Comparison of Innate Immune Signaling Pathways in Primates
Source: PLoS Genet. 2010 Dec 16;6(12):e1001249. doi: 10.1371/journal.pgen.1001249 (PMC3002988; doi:10.1371/journal.pgen.1001249)
Supplement: Table S14 — Primer sequences and PCR conditions for the genes used to validate the immune response to the treatment. (0.03 MB DOC) [file pgen.1001249.s030.doc]

| **Gene** | **Forward primer** | **Reverse primer** |
| --- | --- | --- |
| *IL6* | TACATCCTCGACGGCATCTCA | CTGCAGGAACTGGATCAGGA |
| *IL1B* | AGGAAGATGCTGGTTCCCTG | CATATGGACCAGACATCACC |
| *TNF* | GTTCCTCAGCCTCTTCTCCTTC | TCTGATGGCACCACCAGCTG |
| PCR conditions: | 95°C: 3' |  |
|  | (95°C: 10'', 55°C: 30'',72°C: 30'')X35 |  |
