# Supplementary material for: Functional Comparison of Innate Immune Signaling Pathways in Primates
Source: PLoS Genet. 2010 Dec 16;6(12):e1001249. doi: 10.1371/journal.pgen.1001249 (PMC3002988; doi:10.1371/journal.pgen.1001249)
Supplement: Table S15 — Overlap between the top-ranked genes that responded to the treatment in the different species. We considered the N genes (100, 200, 300, 400, 500, or 1000) showing the largest absolute changes in expression levels following the treatment for each species, and then compared the sets of top ranked genes among species. We report the fold-enrichment for overlap in the paired datasets, relative to that expected by chance (given the number of expressed genes at that time point in each species). (0.03 MB DOC) [file pgen.1001249.s031.doc]

|  | | | | | **Fold-enrichment** | | | | |
| --- | --- | --- | --- | --- | --- | --- | --- | --- | --- |
|  | **Human vs Chimps (4H)** | **Human vs Rhesus (4H)** | **Chimps vs Rhesus (4H)** | **Human vs Chimps (12H)** | **Human vs Rhesus (12H)** | **Chimps vs Rhesus (12H)** | **Human vs Chimps (24H)** | **Human vs Rhesus (24H)** | **Chimps vs Rhesus (24H)** |
| **Top 100 genes** | 826.51 | 3466.06 | 542.49 | 2974.68 | 2974.68 | 1948.93 | 2279.25 | 1734.76 | 1422.19 |
| **Top 200 genes** | 206.63 | 789.06 | 112.44 | 669.77 | 626.44 | 626.44 | 541.98 | 640.80 | 420.49 |
| **Top 300 genes** | 77.69 | 330.52 | 49.97 | 282.67 | 312.88 | 274.18 | 240.88 | 326.09 | 192.75 |
| **Top 400 genes** | 45.68 | 189.68 | 33.91 | 153.04 | 189.68 | 145.96 | 147.72 | 180.32 | 116.75 |
| **Top 500 genes** | 33.83 | 114.22 | 27.71 | 99.32 | 129.68 | 92.07 | 92.07 | 122.84 | 74.51 |
| **Top 1000 genes** | 10.80 | 27.85 | 9.43 | 26.91 | 33.16 | 23.24 | 24.26 | 34.79 | 23.35 |
