# Supplementary material for: Functional Comparison of Innate Immune Signaling Pathways in Primates
Source: PLoS Genet. 2010 Dec 16;6(12):e1001249. doi: 10.1371/journal.pgen.1001249 (PMC3002988; doi:10.1371/journal.pgen.1001249)
Supplement: Table S16 — Consistent enrichment of apoptosis and cancer related genes among human-specific response genes regardless of the cutoffs (within a considerable range) used to define differently expressed genes. (0.03 MB DOC) [file pgen.1001249.s032.doc]

| **Cutoffs Used** | | | | | | **Enrichment *P*-values** | |
| --- | --- | --- | --- | --- | --- | --- | --- |
| FDR Human | FDR Chimpanzees | FDR Rhesus macaques | LPS effect size Humans | LPS effect size Chimpanzees | LPS effect size Rhesus macaques | Apoptosis genes among human-specific responses | Genes involved in cancer pathways among human-specific responses |
| 0.001 | 0.001 | 0.001 | 0 | 0 | 0 | **0.0154148** | **≤0.00155993** |
| 0.001 | 0.01 | 0.001 | 0 | 0 | 0 | **0.0113439** | **≤0.00139179** |
| 0.001 | 0.01 | 0.001 | 0.3 | 0.1 | 0.3 | **0.00832977** | **≤0.00169896** |
| 0.0001 | 0.0001 | 0.0001 | 0 | 0 | 0 | >0.05 | **≤0.0046577** |
| 0.0001 | 0.01 | 0.0001 | 0 | 0 | 0 | **0.0425496** | **≤0.00472276** |
| 0.0001 | 0.01 | 0.0001 | 0.3 | 0.1 | 0.3 | **0.0265432** | **≤0.0156289** |
