# Supplementary material for: Functional Comparison of Innate Immune Signaling Pathways in Primates
Source: PLoS Genet. 2010 Dec 16;6(12):e1001249. doi: 10.1371/journal.pgen.1001249 (PMC3002988; doi:10.1371/journal.pgen.1001249)
Supplement: Text S1 — Results from a control experiment testing for possible biases due to the choice of monocyte purification approach. (0.04 MB DOC) [file pgen.1001249.s033.doc]

**Text S1**

We performed a control experiment to test for possible biases due to the choice of monocyte purification approach. To do so, we obtained whole blood from three additional human samples, from which we purified monocytes using both a positive and a negative selection method. We then performed the same LPS stimulation experiment described for the main samples from the three species, and compared differences in response to LPS treatment between monocytes purified by positive selection and monocytes purified by negative selection. Since we were working only with human samples, in this case we used the Illumina BeadChip HumanHT-12 arrays instead of the customized multi-species arrays, which are significantly more expensive and unnecessary for the purpose of this control experiment (79% of genes analyzed with the multi-species array were also analyzed in the control experiment). As illustrated in the Figure S15, we observed a very strong correlation between the LPS responses of monocytes purified by either positive or negative selection, regardless of the time-point (R^2^ ≥ 0.82). The observed correlations in response to LPS treatment are much higher than those observed between any of the inter-species pairwise comparisons, which did not exceed an R^2^ of 0.45 (see Figure S3).

Using the same statistical cutoffs as those described for our main analysis, we observed that *91%* of the genes classified as differently expressed after LPS treatment, at any time-point, responded to treatment in both monocytes purified by positive selection and monocytes purified by negative selection. This overlap is an underestimate because, due to a smaller sample size (n = 3), we have less power to detect the response phenotypes in the control experiment. Nonetheless, The observed overlap is far greater than any overlap observed in our inter-species comparisons (see Figure 1 of the paper).

Moreover, the few genes that appeared to have responded to the treatment in either positively or negatively selected monocytes, but not in both, were not enriched among genes that were classified as responding to stimulation with LPS differently in humans and chimpanzees (χ^2^ test; *P*= 0.76), or in chimpanzee and rhesus macaques (χ^2^ test; *P*= 0.63). This observation strongly implies that the differences in immune response observed between either humans or rhesus on the one hand, and chimpanzee on the other hand, cannot be generally accounted for by differences in the method utilized to purify monocytes

Finally, we specifically tested whether the set of genes that responded to LPS only in monocytes purified by negative section includes genes classified as responding to LPS treatment exclusively in chimpanzees. Of the 64 genes that responded to LPS treatment only in monocytes purified by negative selection, only 2 are included in our list of chimpanzee-specific responses (*P of overlap* = 0.41). This result is robust with respect to the cutoff used to classify significant regulatory responses, as illustrated in the Figure S16. From a different perspective, only 2 out of 225 genes (0.8%) classified as chimpanzee-specific responses, and for which we had information using the Illumina array (corresponding to 81% of the total number of chimpanzee-specific response genes), were identified as responding to LPS uniquely in the negatively selected monocytes. Neither of these genes (*CCT7* and *TMBIM4*) is part of the set of HIV-1 interacting genes, and in any case, since the overlap of two genes is expected by chance alone, it does not provide strong support for a technical explanation of the observation of chimpanzee-specific pattern even for these two genes.

Taking together, these data clearly demonstrates that the method used to purify monocytes (whether negative or positive selection) has only a minimal effect on the measured regulatory response of the cell cultures to LPS stimulation. However, to be conservative, we excluded from all analyses presented in the manuscript the genes identified as responding to LPS treatment only in monocytes purified by either positive or negative selection.
